# From Chronic Inflammation to Malignancy: Molecular Mechanisms and Therapeutic Insights in Oral Carcinogenesis

**DOI:** 10.3390/ijms27125632

**Published:** 2026-06-22

**Authors:** Ying-Jia Huang, Gaiping Shi, Fengyuan Lv, Ronghua Deng, Qingfeng Zhan, Zixuan Zhang, Jiangyuan Song, Zhi Xu

**Affiliations:** 1 Department of Stomatology, Union Hospital, Tongji Medical College, Huazhong University of Science and Technology, Wuhan 430022, China; m202490008@hust.edu.cn (Y.-J.H.); shigaiping87@163.com (G.S.); m202472798@hust.edu.cn (R.D.); m202572812@hust.edu.cn (Q.Z.); 2 School of Stomatology, Tongji Medical College, Huazhong University of Science and Technology, Wuhan 430030, China; lvfengyuan0825@163.com (F.L.); u202513124@hust.edu.cn (Z.Z.); 3 Hubei Province Key Laboratory of Oral and Maxillofacial Development and Regeneration, Wuhan 430022, China; 4 Center of Stomatology, Tongji Hospital, Tongji Medical College, Huazhong University of Science and Technology, Wuhan 430030, China

**Keywords:** oral cancer, inflammation, precancerous lesions, oral potentially malignant disorders, molecular mechanisms, signaling pathways, biomarkers, precision prevention

## Abstract

Oral squamous cell carcinoma (OSCC) frequently develops within chronically injured oral mucosa and may be preceded by clinically recognizable oral potentially malignant disorders (OPMDs), which provide an important window for cancer interception. This review examines how etiological exposures, persistent inflammation, and lesion-specific epithelial–stromal–immune interactions cooperate during the transition from mucosal injury to dysplasia, carcinoma in situ, and invasive OSCC. Major carcinogenic exposures, including tobacco, alcohol, and areca nut, are considered together with context-dependent contributors such as microbial dysbiosis, viral infection, and immune-mediated epithelial injury. At the molecular level, inflammation-driven oral carcinogenesis involves cytokine and chemokine amplification, oxidative and nitrosative stress, NF-κB and STAT3 activation, the COX-2/PGE_2_ axis, genomic instability, field cancerization, epithelial–stromal crosstalk, angiogenesis, immune dysregulation, and epigenetic and non-coding RNA-mediated reprogramming. Emerging tools such as molecular risk assessment, liquid biopsy, optical imaging, spatially resolved profiling, and artificial intelligence-assisted models may improve identification of high-risk lesions, although most biomarkers require further prospective validation. Prevention should therefore integrate exposure control, biopsy-based diagnosis, local treatment when indicated, long-term surveillance, and trial-based precision strategies according to lesion risk, intervention window, and safety profile. This review supports a shift from lesion-centered management toward risk-adapted precision prevention in inflammation-driven oral carcinogenesis.

## 1. Introduction

Oral squamous cell carcinoma (OSCC), the predominant malignancy of the oral cavity, accounts for more than 90% of oral cancers. In 2020, over 377,000 cases were reported globally, and its burden is projected to rise by nearly 40% by 2040 [1]. Despite technological advances in diagnosis and treatment, the overall five-year survival rate remains disappointingly low—between 40% and 50%—highlighting the urgent need for better prevention and early intervention strategies [2].

**Figure 1:**
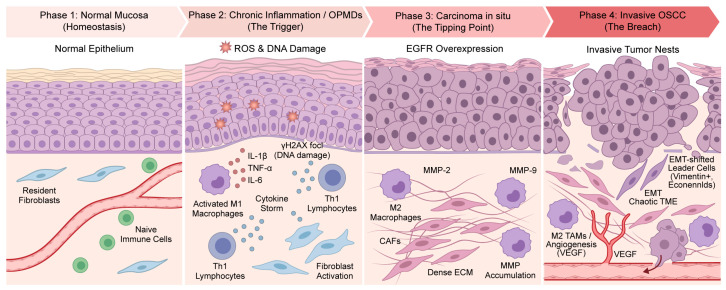
A schematic illustration of inflammation-associated oral carcinogenesis from normal oral mucosa to OPMDs, carcinoma in situ, and invasive OSCC. Normal oral epithelium maintains structural integrity and tissue homeostasis. Persistent exposure to inflammatory and carcinogenic stimuli may induce chronic mucosal inflammation, characterized by immune cell infiltration, cytokine production, oxidative stress, DNA damage, and microenvironmental remodeling. These changes may give rise to clinically recognizable OPMDs, which represent heterogeneous intermediate mucosal conditions with variable biological behavior and malignant transformation risk. With progressive epithelial dysregulation, selected high-risk lesions may evolve into carcinoma in situ, in which atypical epithelial cells remain confined above the basement membrane. Further accumulation of molecular alterations, stromal remodeling, angiogenesis, epithelial–mesenchymal transition, and immune microenvironmental changes may eventually lead to basement membrane disruption, stromal invasion, invasive OSCC, and metastatic spread.

Unlike many malignancies that arise de novo, OSCC develops through a continuum, beginning with chronic injury to the oral epithelium. This persistent damage—often initiated by well-known lifestyle and environmental factors such as tobacco, alcohol, betel nut, and human papillomavirus (HPV) infection—triggers chronic inflammation, creating a pro-tumorigenic environment long before a malignant cell ever forms [3,4]. Over time, the inflamed epithelium may undergo structural and genetic changes that manifest clinically as oral potentially malignant disorders (OPMDs), including oral leukoplakia (OLK), oral erythroplakia (OE), oral submucous fibrosis (OSF), and oral lichen planus (OLP)—each with distinct etiological backgrounds, clinicopathological characteristics, and malignant transformation risks [5]. Recent systematic reviews and meta-analyses indicate that malignant transformation rates vary substantially among OPMD subtypes and across study designs. The pooled malignant transformation rate has been estimated at approximately 7.2–9.8% for OLK, approximately 19.9% for OE, approximately 4.2–6.0% for OSF, and approximately 1.4% for OLP, with higher risks reported in lesions with epithelial dysplasia, non-homogeneous clinical morphology, high-risk anatomical sites, persistent carcinogenic exposure, or stricter diagnostic criteria [6,7,8,9,10,11]. These values should be interpreted as approximate evidence-based estimates rather than fixed risks, because malignant transformation rates are influenced by diagnostic criteria, baseline exclusion of carcinoma, follow-up duration, geographic exposure patterns, and lesion subtype. Together, these OPMDs represent clinically recognizable intermediate states within inflammation-associated oral carcinogenesis, in which persistent mucosal injury and inflammatory remodeling may promote progression from epithelial dysregulation to carcinoma in situ and, ultimately, invasive OSCC (Figure 1).

This review follows that biological journey—from the initial spark of chronic inflammation to the emergence of malignancy. We explore the molecular and cellular mechanisms that govern this transformation, focusing on the key signaling pathways that drive tumorigenesis, the reprogramming of the immune microenvironment, and the hallmarks of cancer as they emerge in the chronically inflamed oral mucosa. Because OPMDs arise from heterogeneous etiological backgrounds, a mechanistic review of inflammation-driven oral carcinogenesis should first clarify how major environmental, microbial, viral, and immune-mediated factors converge on inflammatory signaling networks. By integrating recent insights from molecular biology, immunology, and clinical oncology, we aim to illuminate critical intervention points and provide a foundation for future targeted therapies.

## 2. Etiological Drivers of OSCC and OPMDs: From Environmental Exposure to Inflammatory Signaling

Oral carcinogenesis is shaped by the interaction between environmental carcinogens, chronic mucosal injury, microbial and immune perturbations, and host susceptibility. However, these etiological factors should not be interpreted as equivalent in either causal strength or clinical relevance. Tobacco, alcohol, and betel quid/areca nut are established carcinogenic exposures with strong epidemiological support in oral carcinogenesis [12]. In contrast, microbial dysbiosis and HPV infection appear to play more context-dependent roles in conventional oral cavity carcinogenesis [13,14,15,16]. Current evidence suggests that microbial dysbiosis may amplify local inflammatory signaling and modify lesion behavior, whereas the etiological role of HPV is much more firmly established in oropharyngeal squamous cell carcinoma than in conventional oral cavity OSCC or OPMDs. Similarly, immune-mediated epithelial injury, as observed in OLP, provides a biologically plausible inflammatory model of malignant transformation, but its absolute malignant risk remains lower and more controversial than that of high-risk leukoplakia, erythroplakia, or OSF [11,17]. Therefore, a critical etiological framework should distinguish well-established causal exposures from emerging modifiers of the inflammatory microenvironment. This distinction is clinically important because prevention strategies should prioritize elimination of validated causal exposures while using molecular and immune biomarkers to refine risk in more heterogeneous or uncertain settings.

Tobacco exposure remains one of the most firmly established etiological drivers of OLK, epithelial dysplasia, and OSCC. Tobacco smoke and smokeless tobacco contain multiple carcinogenic compounds, including tobacco-specific nitrosamines, polycyclic aromatic hydrocarbons, and reactive aldehydes, which induce DNA adduct formation, oxidative DNA damage, chromosomal instability, and mutations in key tumor suppressor genes such as TP53 [12]. Beyond direct genotoxicity, tobacco exposure also promotes chronic mucosal inflammation by stimulating epithelial cells, fibroblasts, endothelial cells, and infiltrating immune cells to produce IL-1β, IL-6, TNF-α, COX-2-derived prostaglandins, and other inflammatory mediators [12,18]. These signals converge on NF-κB, STAT3, MAPK, and phosphoinositide 3-kinase (PI3K)–protein kinase B (AKT) pathways, thereby promoting epithelial proliferation, apoptosis resistance, angiogenesis, and immune escape. Importantly, the translational significance of tobacco exposure lies not only in its mechanistic link to carcinogenesis but also in its preventability. Nevertheless, tobacco-related risk cannot be fully reversed by lesion removal alone, because carcinogen-induced molecular alterations may extend beyond clinically visible lesions and contribute to field cancerization, recurrence, and second primary tumors. Thus, tobacco cessation should be regarded as a foundational intervention, but not as a substitute for long-term surveillance in patients with established OPMDs.

Alcohol consumption further contributes to oral carcinogenesis, particularly in combination with tobacco exposure. Ethanol is metabolized to acetaldehyde, a reactive and mutagenic compound capable of forming DNA adducts, impairing DNA repair, and promoting chromosomal instability. Alcohol also increases epithelial permeability, facilitates penetration of tobacco-derived carcinogens, alters local retinoid metabolism, and enhances oxidative stress, thereby amplifying inflammatory signaling within the oral mucosa [19,20]. The synergistic interaction between tobacco and alcohol is clinically important because combined exposure confers a substantially greater risk of OSCC than either exposure alone [12,19]. However, the alcohol-related pathway also illustrates a recurring limitation in oral carcinogenesis research: epidemiological associations are strong, but it is often difficult to separate direct genotoxic effects from indirect inflammatory, nutritional, microbial, and behavioral confounders. Therefore, alcohol should be discussed as both a carcinogenic exposure and an amplifier of epithelial vulnerability, especially in patients with concurrent tobacco use or pre-existing OPMDs.

Betel quid and areca nut chewing represent the clearest example of an exposure with a disease-specific mechanistic link to an OPMD, particularly OSF. Areca nut and betel quid are established carcinogenic exposures, and arecoline, a major areca alkaloid, induces oxidative stress, DNA damage, mitochondrial dysfunction, inflammatory cytokine production, and fibroblast activation [12,20]. In OSF, arecoline-driven activation of TGF-β/Smad signaling promotes collagen synthesis, myofibroblast differentiation, extracellular matrix deposition, and suppression of matrix degradation through altered altered balance between matrix metalloproteinases (MMPs)/tissue inhibitors of metalloproteinases (TIMPs) balance [20]. This profibrotic process increases tissue stiffness and may further activate mechanotransduction pathways such as integrin–focal adhesion kinase (FAK)–Src and yes-associated protein/transcriptional coactivator with PDZ-binding motif (YAP/TAZ) signaling, thereby linking stromal remodeling to epithelial dysregulation. Compared with other OPMDs, OSF therefore provides a relatively coherent exposure–inflammation–fibrosis–carcinogenesis axis. However, the contribution of individual mechanisms to malignant transformation remains difficult to quantify because many patients have combined exposure to areca nut, tobacco, alcohol, and nutritional deficiencies. This limitation should temper deterministic interpretations of OSF progression while reinforcing the clinical priority of areca nut cessation and early fibrosis control.

Persistent microbial dysbiosis and chronic mucosal infection have attracted increasing attention as contributors to inflammation-driven oral carcinogenesis. Dysbiotic microbial communities may activate Toll-like receptor signaling, inflammasome-related pathways, NF-κB, STAT3, and cytokine networks, thereby promoting epithelial barrier dysfunction, IL-1β, IL-6, TNF-α, and IL-17 production, and immune microenvironmental remodeling [13,14]. Certain microorganisms may also contribute to carcinogenesis by producing acetaldehyde, promoting nitrosamine formation, modulating oxidative stress, or interfering with epithelial immune surveillance [14]. Nevertheless, the microbiome should currently be interpreted more cautiously than tobacco, alcohol, or areca nut. Most available studies are cross-sectional or associative, making it difficult to determine whether microbial dysbiosis is a cause, consequence, or modifier of OPMD progression. The most defensible interpretation is that microbial dysbiosis may amplify inflammatory signaling and modify lesion behavior, but specific microbial taxa have not yet been validated as independent causal drivers or routine clinical risk markers in OPMDs. Future longitudinal and mechanistic studies are required before microbiome-based diagnostics or therapies can be integrated into standard OPMD management.

Viral infection, particularly HPV, requires careful anatomical and biological distinction. High-risk HPV types, especially HPV16, promote malignant transformation through E6- and E7-mediated disruption of p53 and retinoblastoma protein function, leading to impaired apoptosis, cell cycle deregulation, and genomic instability. However, HPV-driven carcinogenesis is much more firmly established in oropharyngeal squamous cell carcinoma than in conventional oral cavity OSCC or OPMDs [15,16]. Therefore, HPV should not be presented as a dominant universal driver of oral cavity carcinogenesis. Instead, it should be framed as a potential context-dependent contributor whose relevance may vary according to anatomical site, detection method, viral transcriptional activity, and host immune status. This distinction is important because overgeneralizing HPV biology from oropharyngeal cancer to oral cavity OPMDs may lead to misleading mechanistic and clinical assumptions. In the OPMD setting, HPV testing may be informative in selected cases, but its role in routine risk stratification remains less established than conventional clinicopathological factors and validated molecular markers.

Immune-mediated epithelial injury is particularly relevant to OLP and oral lichenoid lesions. In these disorders, chronic T cell-mediated damage to basal keratinocytes creates a persistent cycle of epithelial apoptosis, regeneration, cytokine release, and immune cell recruitment [17,18]. CD8^+^ cytotoxic T cells, T helper 1 (Th1)/T helper 17 (Th17)-skewed responses, interferon-gamma (IFN-γ), TNF-α, IL-17, and chemokine networks contribute to basal cell degeneration and sustained mucosal inflammation [18]. This mechanism provides a biologically plausible link between chronic immune injury and epithelial instability. However, the malignant potential of OLP remains more controversial than that of leukoplakia, erythroplakia, or OSF, partly because diagnostic criteria, inclusion of oral lichenoid lesions, epithelial dysplasia at baseline, and follow-up duration vary across studies [17]. Therefore, OLP should be described as a chronic immune-mediated disorder with low but non-negligible malignant potential, rather than as a uniformly high-risk premalignant lesion. Clinically, risk assessment in OLP should emphasize lesion subtype, persistence of erosive or ulcerative disease, histopathological changes, and coexisting carcinogenic exposures.

Taken together, the etiological landscape of OSCC and OPMDs supports a risk-adapted rather than exposure-neutral model of oral carcinogenesis. Tobacco, alcohol, and areca nut represent high-priority causal exposures because they combine strong epidemiological evidence, mechanistic plausibility, and actionable prevention strategies [12,19,20,21]. In contrast, microbial dysbiosis, HPV infection, and immune-mediated injury should be interpreted as context-dependent modifiers whose clinical utility depends on lesion subtype, anatomical site, host immune status, and validation of disease-specific biomarkers [11,13,14,15,16,17]. These etiological factors converge on shared biological processes, including oxidative and nitrosative stress, epithelial barrier disruption, DNA damage, cytokine and chemokine amplification, stromal remodeling, immune escape, and epigenetic reprogramming. However, convergence at the pathway level does not imply identical clinical management. The central translational challenge is to determine which etiological drivers are removable, which molecular alterations are reversible, and which lesions require intensified surveillance or intervention. This critical distinction provides the rationale for the following sections, which examine how chronic inflammation reshapes the oral epithelial and stromal microenvironment and how these mechanisms may be translated into risk-stratified prevention strategies.

## 3. Chronic Inflammation of the Oral Cavity and Tumorigenesis

### 3.1. Inflammatory Cells, Mediators, and Microenvironmental Remodeling

Chronic inflammation initiates and sustains a tumor-promoting microenvironment through the coordinated action of immune cells, cytokines, chemokines, oxidative stress, and stromal remodeling. The persistent presence of inflammatory mediators and infiltrating immune cells not only promotes epithelial damage but also alters the biological landscape in favor of malignant transformation. However, chronic inflammation should not be interpreted as a single linear carcinogenic stimulus. Rather, it acts as a dynamic tissue-level process in which epithelial injury, immune cell recruitment, fibroblast activation, extracellular matrix remodeling, oxidative DNA damage, and immune suppression reinforce one another over time [22]. This distinction is important because the same inflammatory pathway may serve different functions at different stages of oral carcinogenesis: early inflammation may reflect host defense or tissue repair, whereas persistent unresolved inflammation may promote epithelial instability, field cancerization, and malignant progression.

#### 3.1.1. Cytokine Networks, Chemokines, and Inflammatory Mediators

The pro-inflammatory cytokine network within the oral chronic inflammatory microenvironment plays a central role in inflammation-to-cancer transformation. The expression of pro-inflammatory factors, such as interleukin (IL)-1β, IL-6, tumor necrosis factor-α (TNF-α), and IL-17, is upregulated, constituting a mutually reinforcing regulatory loop that not only maintains a persistent inflammatory state but also directly participates in the process of malignant transformation [23].

IL-6, produced mainly by activated macrophages, fibroblasts, and epithelial cells, is a central mediator linking inflammation and tumorigenesis. It regulates genes associated with proliferation, differentiation, survival, angiogenesis, and immune modulation by binding to the gp130 receptor and activating the Janus kinase (JAK)–signal transducer and activator of transcription 3 (STAT3) pathway [24,25]. IL-6 can also upregulate cyclooxygenase-2 (COX-2) expression and increase prostaglandin E2 (PGE_2_) synthesis, thereby participating in a positive inflammatory feedback loop [26]. Nevertheless, IL-6 should be interpreted not only as a single soluble marker but also as part of a broader cytokine circuit that connects epithelial stress, stromal activation, and immune suppression. This is clinically relevant because elevated cytokine expression alone does not establish therapeutic vulnerability unless pathway dependence and treatment responsiveness are demonstrated.

TNF-α promotes epithelial proliferation, apoptosis resistance, oxidative stress, and epithelial–mesenchymal transition (EMT) through activation of the nuclear factor-κB (NF-κB), mitogen-activated protein kinase (MAPK) pathways, and other stress-responsive pathways. TNF-α may also induce reactive oxygen species (ROS) production, which leads to oxidative DNA damage, and upregulates epithelial–mesenchymal transition (EMT)-associated transcription factors such as Snail and Twist, thereby promoting OSCC invasive ability [27,28,29,30,31,32,33]. IL-1β is positively correlated with COX-2 expression in oral precancerous lesion tissues, and the two synergistically promote angiogenesis and tumor invasion [34]. IL-17, produced mainly by infiltrating Th17 cells, is significantly elevated in OLP and early OSCC; studies have demonstrated that IL-17 promotes tumor progression through activation of the NF-κB and MAPK pathways, facilitating the activation of cancer-associated fibroblasts (CAFs) and creating an immunosuppressive microenvironment [35,36,37,38,39].

In addition to classical inflammatory cytokines, chemokines contribute to the spatial organization and cellular composition of the inflammatory microenvironment. C-C motif chemokine ligand 2 (CCL2), also known as monocyte chemotactic protein-1 (MCP-1), primarily recruits CCR2-positive monocytes and macrophages to sites of inflammation and neoplastic tissues. In OSCC and head and neck squamous cell carcinoma, elevated MCP-1/CCL2 expression has been associated with tumor progression, macrophage accumulation, pro-survival signaling, and poor clinical outcome [40]. Therefore, MCP-1/CCL2 may serve as a link between inflammatory chemokine signaling and tumor-promoting macrophage recruitment. However, its independent value as a prognostic or risk stratification biomarker in OPMDs remains insufficiently validated and should be interpreted cautiously.

Collectively, cytokine and chemokine networks do not merely reflect inflammation; they actively shape the oral lesion microenvironment by regulating immune cell recruitment, epithelial survival, fibroblast activation, angiogenesis, and immune escape. The critical translational issue is to determine which cytokine circuits are drivers of progression and which are secondary markers of tissue injury. This distinction is essential for avoiding overinterpretation of inflammatory biomarkers as therapeutic targets.

#### 3.1.2. Oxidative Stress and DNA Damage

Oxidative stress is a key mechanism by which chronic inflammation promotes tumorigenesis. In the oral inflammatory microenvironment, infiltrating neutrophils and macrophages produce large amounts of ROS and reactive nitrogen species (RNS) via NADPH oxidase, myeloperoxidase (MPO), and inducible nitric oxide synthase (iNOS), disrupting the oxidative/antioxidant balance [41]. PGE_2_ enhances oxidative stress through multiple mechanisms, including increased NADPH oxidase expression and inhibition of antioxidant enzyme activities [42]. Kaur et al. demonstrated that salivary 8-hydroxydeoxyguanosine (8-OHdG) and malondialdehyde (MDA) levels are significantly elevated in patients with OLP, OLK, OSF, and OSCC compared to healthy controls, suggesting that salivary oxidative stress biomarkers may serve as diagnostic indicators across the oral premalignant-to-malignant spectrum [43]. Concurrently, the activities of antioxidant defense systems such as superoxide dismutase (SOD), glutathione peroxidase (GPx), and catalase (CAT) decrease, further exacerbating this imbalance [44]. This imbalance is significantly and positively correlated with the risk of malignancy in oral precancerous lesions [45].

Oxidative stress promotes carcinogenesis through several interconnected mechanisms. First, ROS directly induce DNA base modifications, strand breaks, and mutations, activating the MAPK cascade and upregulating cell cycle proteins such as Cyclin D1. Second, oxidative inactivation of the tumor suppressor PTEN relieves its inhibitory effect on phosphoinositide 3-kinase (PI3K), thereby activating the PI3K/AKT survival pathway and promoting cell proliferation and resistance to apoptosis. Third, sustained ROS generation activates the NF-κB pathway and upregulates anti-apoptotic proteins including Bcl-2 and Bcl-XL [46]. Mitochondria, as both the primary source and a critical target of ROS, undergo functional impairment that further amplifies ROS generation in a self-reinforcing positive feedback loop.

Importantly, oxidative stress should be interpreted as both a mechanism of injury and a marker of an unstable inflammatory field. Elevated oxidative biomarkers may indicate tissue damage and increased carcinogenic pressure, but they do not automatically justify high-dose antioxidant supplementation as chemoprevention. This distinction is clinically important because antioxidant interventions that appear protective in mechanistic models may produce neutral or even harmful effects in high-risk human populations, particularly among tobacco-exposed individuals. Therefore, oxidative stress provides strong mechanistic support for inflammation-driven carcinogenesis, but antioxidant therapy requires separate evidence of clinical benefit and safety.

#### 3.1.3. Remodeling of the Inflammatory Microenvironmental and Immune Landscape Reprogramming

Oral inflammation-induced microenvironmental remodeling is a decisive component of the inflammatory-to-cancer transformation, encompassing extracellular matrix (ECM) remodeling, immune microenvironmental alterations, and the EMT process. In chronic inflammatory states, the balance between MMPs and TIMPs is disrupted, and the activities of MMP-2, MMP-9, and MMP-13 are significantly elevated, degrading basement membranes and ECM components [47]. Quan et al. demonstrated that MMP-9 expression in precancerous lesion tissues is positively correlated with the risk of malignant transformation and can serve as a predictive marker [48]. Activated fibroblasts transform into myofibroblasts that secrete large amounts of collagen fibers, leading to tissue stiffness [49]. This altered stiffness promotes proliferation and EMT through integrin-FAK-Src signaling, activation of the YAP/TAZ and transforming growth factor beta (TGF-β) pathways, and is particularly evident during OSF malignancy [50].

Reprogramming of the immune microenvironment reflects the shift from an inflammatory to a tumor-promoting phenotype. An increased proportion of regulatory T cells (Tregs), accumulation of M2-polarized macrophages, and expansion of myeloid-derived suppressor cells (MDSCs) together create an immunosuppressive milieu [51]. Mori, K. et al. found that the number of CD163^+^ M2-type macrophages infiltrating oral precancerous lesion tissues was significantly correlated with the risk of malignant transformation [52]. STAT3 activation plays a key role in suppressing immune cell function, making it an important target for immunotherapy [53].

TGF-β, IL-6, and TNF-α promote epithelial–mesenchymal transition (EMT), a critical process through which epithelial cells acquire invasive and migratory capacities. EMT is characterized by the downregulation of epithelial markers such as E-cadherin and the upregulation of mesenchymal markers including vimentin, mediated by key transcription factors such as Snail, Twist, and ZEB1 [54]. Accumulating evidence indicates that EMT marker expression in oral precancerous lesions correlates closely with the degree of epithelial dysplasia and the risk of malignant transformation [55,56].

In parallel, inflammatory signaling drives vascular remodeling within the lesion microenvironment. The upregulation of vascular endothelial growth factor A (VEGF-A) and VEGF-C promotes angiogenesis and lymphangiogenesis, respectively, thereby facilitating tumor growth and metastatic dissemination [57]. These processes should be interpreted as interconnected rather than independent events. For example, fibroblast activation and matrix stiffening may enhance epithelial mechanotransduction; immune suppression may permit survival of genetically altered epithelial clones; and angiogenesis may support the metabolic demands of expanding dysplastic tissue. Together, these interactions create a permissive microenvironment that enables the transition from localized epithelial dysregulation to carcinoma in situ and invasive carcinoma.

A critical implication is that the inflammatory microenvironment is not only a background condition but also a selective pressure. It can favor the expansion of epithelial clones that tolerate oxidative stress, evade immune surveillance, respond to growth signals, and invade remodeled stroma. Therefore, assessment of OPMD progression should not be limited to epithelial dysplasia alone; stromal, immune, and vascular features may provide additional information about biological risk.

### 3.2. Key Inflammatory Signal Transduction in Oral Carcinogenesis

**Figure 2:**
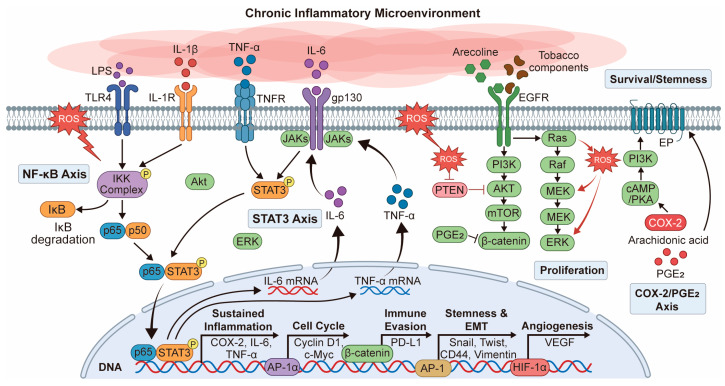
From OPMDs to OSCC: the integrated inflammatory signaling networks. A schematic representation of interconnected signaling pathways activated within the chronic inflammatory microenvironment of OPMDs. Extracellular inflammatory mediators, microbial components, carcinogens, and ROS engage multiple membrane receptors and initiate parallel intracellular signaling cascades, including the NF-κB and STAT3 axis, EGFR-dependent PI3K–AKT–mTOR and MAPK pathways, and the COX-2/PGE_2_ signaling axis. Extensive crosstalk among these pathways enables coordinated transcriptional regulation of gene programs associated with inflammation, cell proliferation, immune modulation, EMT, stemness, and angiogenesis, collectively contributing to a pro-tumorigenic state during oral carcinogenesis.

Signal transduction pathways activated by inflammatory stimuli play a pivotal role in linking chronic inflammation with carcinogenesis. Among these, NF-κB, STAT3, and the COX-2/PGE_2_ axis have been extensively characterized as central regulators of inflammation-driven oral tumorigenesis. These pathways interact with EGFR-dependent PI3K–AKT–mTOR and MAPK signaling, ROS-mediated stress responses, and prostaglandin signaling to coordinate transcriptional programs related to sustained inflammation, proliferation, survival, immune evasion, EMT, stemness, and angiogenesis (Figure 2).

However, these pathways should not be interpreted as redundant or therapeutically equivalent. Although they converge on overlapping cancer-associated phenotypes, they differ in their upstream triggers, dominant biological functions, cellular compartments, and translational druggability. NF-κB is best understood as a broad inflammatory amplifier, STAT3 as an inflammation–immunity–survival integrator, and COX-2/PGE_2_ as a prostaglandin-mediated effector axis with a well-recognized but clinically constrained chemopreventive rationale. This distinction is important because pathway activation does not automatically imply that direct pathway inhibition is clinically feasible or beneficial in OPMDs.

#### 3.2.1. NF-κB Signaling Pathway

NF-κB is a pivotal transcription factor linking inflammation and cancer, comprising the subunits RelA (p65), RelB, c-Rel, p50, and p52. The NF-κB pathway is activated in response to a variety of inflammatory signals, including TNF-α, IL-1β, Toll-like receptor (TLR) ligands, and oxidative stress [58]. In the oral inflammatory microenvironment, persistent NF-κB activation regulates multiple aspects of cell proliferation, apoptosis, inflammatory response, and microenvironmental remodeling. NF-κB promotes tumorigenesis through multiple mechanisms: firstly, upregulation of anti-apoptotic proteins such as Bcl-2 and Bcl-XL, allowing DNA-damaged cells to evade apoptotic checkpoints [59]; secondly, upregulation of Cyclin D1, CDK2/4, and other cell cycle proteins to promote G1/S phase transition [60]; thirdly, the upregulation of human telomerase reverse transcriptase (hTERT) expression to extend telomere length and confer unlimited proliferative capacity [61]; and fourthly, upregulation of pro-inflammatory genes such as COX-2 and IL-6, forming a self-amplifying cycle of inflammation [62]. Through the regulation of anti-apoptotic genes, cell cycle regulators, matrix-remodeling enzymes, angiogenic factors, and immune-modulatory molecules, NF-κB contributes to epithelial survival, proliferation, stromal invasion, and immune escape [63].

Within the tumor microenvironment, NF-κB promotes VEGF and IL-8 expression to enhance angiogenesis, upregulates MMPs to promote stromal remodeling and invasion, and induces EMT to enhance migratory capacity [64,65]. NFκB also promotes immune escape through the upregulation of immunosuppressive factors such as PD-L1 and IL-10. Recent studies have shown that NF-κB interacts with transcription activation domain (TAD) proteins to play a key role in oral cancer stem cell maintenance [66].

From normal oral mucosa through inflammation and precancerous lesions to OSCC, the level of NF-κB nuclear localization and activation increases progressively, correlating with disease progression and prognosis [67,68,69,70]. NF-κB pathway activation, as reflected by p65 nuclear translocation, has been implicated in oral carcinogenesis [68]. Immunohistochemical studies have demonstrated that p-AKT, an upstream activator of NF-κB, shows significantly higher nuclear expression in oral epithelial dysplasia compared to normal mucosa, with levels progressively increasing in OSCC [71]. Moreover, the proliferation marker Ki-67 exhibits a stepwise increase from normal mucosa through precancerous lesions to OSCC [72]. These findings suggest that the NF-κB signaling axis may be associated with both histological dysplasia grade and cellular proliferation, warranting further multicenter validation [73].

#### 3.2.2. STAT3 Signaling Pathway

STAT3 is a key effector molecule of the JAK-STAT pathway and plays a pro-oncogenic role in a variety of tumors. STAT3 is primarily activated by the IL-6 family of cytokines, growth factors, and cytokine receptor-associated kinases [74]. In the oral inflammatory microenvironment, IL-6 binds to the gp130 receptor to activate JAKs, leading to phosphorylation of STAT3 at Tyr705, which promotes dimerization, nuclear translocation, and transcription of target genes [75]. STAT3 regulates a variety of cancer-related functions: upregulation of oncogenes such as Cyclin D1 and c-Myc, and downregulation of anti-oncogenes, such as p53 and p21, to promote cell proliferation [76]; upregulation of anti-apoptotic proteins such as Survivin and Bcl-2 to enhance cell survival [77]; and upregulation of angiogenic factors such as VEGF and bFGF to promote angiogenesis [78]. Recent studies have shown that STAT3 plays an important role in radiotherapy and chemotherapy resistance in oral cancer [79].

STAT3 has been described as a key regulator of immune checkpoint signaling [80], inhibiting the function of CD8^+^ T cells and natural killer (NK) cells, promoting the expansion of MDSCs and Tregs, and creating an immunosuppressive environment. In addition, STAT3 promotes invasion and metastasis by upregulating EMT transcription factors such as Snail and Twist [81]. Recent single-cell sequencing analysis revealed that STAT3 plays a key role in shaping the oral cancer immune microenvironment [82]. NF-κB and STAT3 display extensive functional crosstalk, forming the “NF-κB–IL-6–STAT3” signaling loop: NF-κB activation upregulates IL-6 expression to activate STAT3; reciprocally, STAT3 maintains NF-κB activation, and the two form a transcriptional complex that synergistically regulates specific target genes [83]. This mutually reinforcing relationship amplifies inflammatory and oncogenic signals, forming a self-sustaining cycle that is a central driver of oral inflammation-to-cancer transformation [84].

Compared with NF-κB, STAT3 may represent a more actionable immunomodulatory node because it integrates epithelial survival with immune suppression. However, most STAT3-targeted strategies remain preclinical or early translational in the OPMD context. Evidence supporting STAT3 as a driver of inflammatory carcinogenesis is stronger than evidence supporting STAT3 inhibition as a clinically validated preventive intervention. Therefore, STAT3 should be discussed as a promising translational target whose therapeutic relevance depends on lesion-specific pathway activation, immune contexture, delivery strategy, and safety profile. At present, its most realistic value in OPMDs may be as part of molecular risk assessment or as a trial-based target in biologically selected high-risk lesions.

#### 3.2.3. COX-2/PGE_2_ Axis

The COX-2/PGE_2_ axis serves as a pivotal link between inflammation and tumorigenesis. COX-2 is an inducible enzyme that is rapidly upregulated in response to inflammatory stimuli and catalyzes the conversion of arachidonic acid to PGE_2_ [85]. In oral inflammation, IL-1β, TNF-α, and growth factors upregulate COX-2 expression, whereupon PGE_2_ activates multiple downstream signaling pathways via EP1-4 receptors [86]. COX-2/PGE_2_ promotes oral carcinogenesis through multiple mechanisms: first, driving cell proliferation and survival via the EP2/EP4–cAMP–PKA and PI3K–AKT pathways [87]; second, upregulating VEGF to promote angiogenesis [88]; third, inhibiting NK cell and T cell function to promote Treg differentiation and create an immunosuppressive environment [89]; and fourth, inducing EMT and upregulating MMPs to promote invasion and metastasis [90]. In addition, the COX-2/PGE_2_ axis interacts with the stem cell signaling pathways Wnt/β-catenin and Notch to maintain cancer stem cell properties [91]. Long-term use of non-steroidal anti-inflammatory drugs (NSAIDs) and selective COX-2 inhibitors (e.g., celecoxib) has been associated with a reduced risk of oral and head and neck cancers [92]. Meta-analyses have suggested that NSAID use is associated with a modest reduction in the risk of head and neck cancer, although the magnitude and consistency of this effect vary across studies [93]. Animal studies have demonstrated that COX-2 inhibition attenuates chemically induced oral precancerous lesions and delays malignant progression [94].

COX-2 expression increases in a stepwise fashion from normal oral mucosa through inflammation and precancerous lesions to OSCC [95]. A multicenter study showed that high COX-2 expression in oral precancerous lesions was significantly correlated with the degree of epithelial dysplasia, lesion size, and the risk of malignancy [96]. Combined detection of COX-2 with molecular markers such as EGFR and p53 improves the accuracy of malignant risk assessment [97,98]. Clinical studies have further demonstrated that PGE_2_ levels in lesional tissues can be modulated by COX-2 inhibitors, suggesting that PGE_2_ represents a key driver of oral premalignant disease [99,100]. These findings raise the possibility that salivary PGE_2_ measurement could serve as a noninvasive biomarker to monitor disease activity, although direct validation in saliva samples is warranted.

However, the COX-2/PGE_2_ axis also illustrates the gap between mechanistic plausibility and clinical chemoprevention. Although COX-2 inhibition can suppress oral carcinogenesis in experimental models, clinical evidence in oral premalignant lesions has not established sufficient benefit for routine preventive use. A randomized phase II trial of celecoxib in oral premalignant lesions did not demonstrate adequate clinical efficacy at the tested doses [101]. Moreover, long-term preventive use of selective COX-2 inhibitors is constrained by cardiovascular toxicity concerns, as demonstrated in large adenoma-prevention trials [102]. Therefore, COX-2 should not be presented as a clinically validated chemopreventive target in OPMDs. Rather, it should be interpreted as a biologically informative inflammatory axis that may support biomarker development, short-term pharmacodynamic studies, or future localized intervention strategies.

Taken together, the NF-κB, STAT3, and COX-2/PGE_2_ pathways form an interconnected inflammatory signaling network that links etiological exposures to epithelial transformation. Their major translational value lies in defining disease biology, identifying high-risk inflammatory states, and guiding evidence-based intervention design. However, pathway activation alone is insufficient to justify therapeutic targeting. Future studies should distinguish pathway association from pathway dependence and should evaluate whether pathway modulation improves clinically meaningful endpoints such as malignant transformation, recurrence, or cancer-free survival.

## 4. Molecular Pathology of Oral Precancerous Lesions

OPMDs encompass a spectrum of clinically and histologically heterogeneous lesions with varying risks of progression to OSCC. Among them, OSF, OLP, and OLK are among the most extensively characterized and illustrate three different but partially convergent models of oral carcinogenesis: fibrosis-dominant transformation, immune-mediated epithelial injury, and epithelial dysplasia-centered progression. Despite their distinct etiologies, these disorders share overlapping pathogenic mechanisms, including chronic inflammation, genetic and epigenetic alterations, epithelial dysregulation, stromal remodeling, and dynamic changes in the immune microenvironment [5].

A unifying concept in understanding their malignant potential is field cancerization. In response to persistent carcinogenic and inflammatory insults, clinically normal-appearing oral mucosa may acquire genomic and epigenomic alterations that predispose to multifocal lesion development, local recurrence, second primary tumors, and malignant transformation [103]. However, the existence of field cancerization does not mean that all OPMDs have equivalent biological behavior or require identical management. Rather, lesion subtype, anatomical site, clinical morphology, histopathological grade, etiological exposure, and molecular risk markers should be interpreted together.

**Figure 3:**
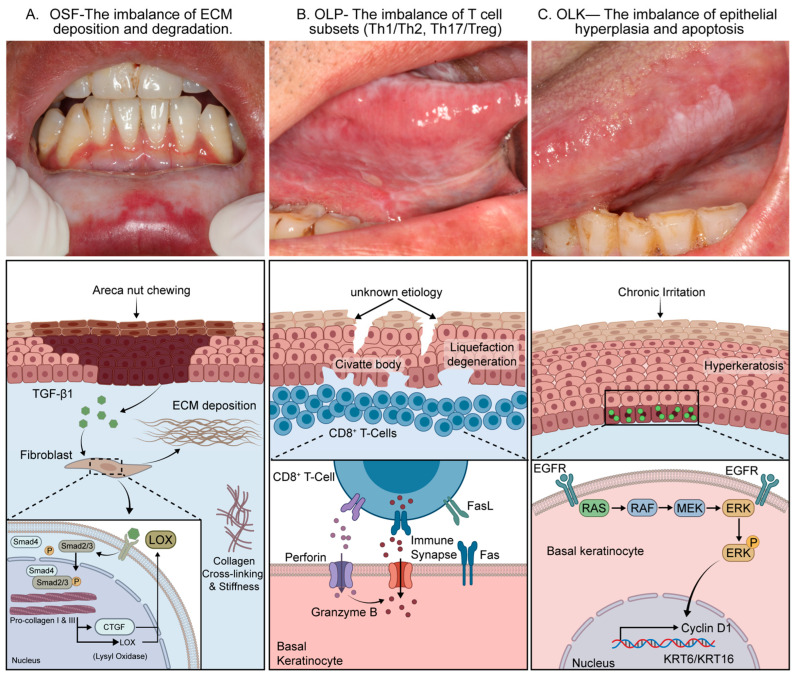
Disease-specific inflammatory and molecular mechanisms in major OPMDs. Representative clinical and schematic comparison of three major OPMDs. In OSF, chronic arecoline exposure and oxidative stress drive fibroblast activation and excessive ECM deposition, representing an imbalance of ECM deposition and degradation. OLP is characterized by T cell-mediated immune responses targeting basal keratinocytes, representing an imbalance of T cell subsets (Th1/Th2 and Th17/Treg). In OLK, epithelial-centered abnormalities predominate, representing an imbalance of epithelial hyperplasia and apoptosis. Despite differing initiating mechanisms, these conditions share sustained inflammatory signaling and increased susceptibility to malignant transformation.

Clinically, OPMDs exhibit distinct phenotypic characteristics that reflect their underlying pathological processes. OLK typically presents as white plaques with variable surface morphology and degrees of epithelial dysplasia, representing epithelial alterations associated with carcinogenic risk. OLP is characterized by reticular or erosive lesions driven by chronic immune-mediated inflammation. In contrast, OSF manifests as mucosal stiffness, blanching, and progressive limitation of mouth opening, reflecting persistent fibrotic remodeling of the oral mucosa. These clinical phenotypes are closely associated with disease-specific inflammatory pathways, stromal interactions, and epithelial responses. Therefore, the molecular pathology of OPMDs should be understood through both shared carcinogenic principles and lesion-specific mechanisms, rather than as a uniform premalignant process (Figure 3).

### 4.1. Oral Submucous Fibrosis (OSF)

#### 4.1.1. Pathologic Features and Molecular Alterations

OSF is a chronic, progressive fibrotic disorder predominantly associated with areca nut (betel quid) chewing. It is characterized by mucosal atrophy, basement membrane thickening, abnormal collagen fiber proliferation, and vitreous degeneration, ultimately leading to tissue sclerosis and restricted mouth opening [104]. Reported malignant transformation estimates for OSF vary across studies and should be interpreted cautiously because available longitudinal studies differ in study population, case definition, follow-up duration, clinicopathological reporting, and assessment of associated risk indicators. Recent systematic reviews and meta-analyses have estimated the pooled malignant transformation proportion of OSF to be approximately 4.2–6.0%, supporting OSF as a potentially malignant, fibrosis-associated disorder that requires long-term surveillance rather than a lesion with a single fixed transformation rate [10,105]. Among major OPMDs, OSF has one of the clearest exposure–mechanism relationships because areca nut exposure, arecoline-induced oxidative stress, TGF-β-driven fibrosis, and stromal stiffening form a coherent pathogenic axis. This makes OSF a useful model for understanding how chemical exposure and fibrotic remodeling cooperate during inflammation-driven oral carcinogenesis.

At the molecular level, OSF is characterized by activation of the TGF-β/Smad pathway, abnormal myofibroblast activation, enhanced oxidative stress, and autophagy dysfunction. The TGF-β/Smad pathway plays a central role: arecoline and mechanical stimulation prompt oral fibroblasts to secrete TGF-β1, which activates Smad2/3 phosphorylation and nuclear translocation [106]. The activated Smad complex initiates transcription of COL1A1, COL1A2, and other matrix protein genes, while upregulating TIMP-1/2 and inhibiting MMP activity, leading to excessive ECM deposition [107]. Non-coding RNAs (ncRNAs) may amplify this profibrotic program; for example, lncRNA-ATB and other OSF-associated lncRNAs have been reported to regulate TGF-β/Smad signaling and fibroblast activation [108,109].

Abnormal myofibroblast activation is a key cellular event in OSF pathogenesis. TGF-β1 drives the transdifferentiation of oral fibroblasts into myofibroblasts, as evidenced by increased expression of α-smooth muscle actin (α-SMA) [110]. These activated myofibroblasts produce excessive extracellular matrix components while generating contractile forces that further enhance TGF-β activation, thereby establishing a self-perpetuating profibrotic feedback loop [111]. Accumulating evidence suggests that the abundance of myofibroblasts in OSF tissues correlates with the degree of fibrosis and disease severity [112]. Importantly, fibrosis should not be viewed as a passive scar-like outcome. Increased ECM deposition, collagen cross-linking, and tissue stiffness may actively reshape epithelial behavior through integrin–FAK–Src, YAP/TAZ, and TGF-β-dependent mechanotransduction pathways. Thus, the malignant potential of OSF is likely related not only to epithelial genotoxic injury but also to the creation of a mechanically altered stromal niche.

Oxidative stress is also crucial in the pathogenesis of OSF. ROS and RNS produced by areca nut constituents can directly damage oral mucosal cells, causing lipid peroxidation, protein oxidation, DNA damage, and mitochondrial dysfunction [113]. Studies have shown that 8-OHdG and MDA levels are significantly elevated in saliva of OSF patients, whereas antioxidant enzyme activities are decreased, supporting the role of oxidative injury in OSF-associated carcinogenesis [43]. Oxidative stress not only directly damages DNA but also upregulates the expression of pro-inflammatory and fibrogenic factors by activating transcription factors such as NF-κB and AP-1 [114]. However, oxidative stress biomarkers should be interpreted cautiously. They reflect tissue injury and carcinogenic pressure, but they do not by themselves establish malignant transformation risk or justify antioxidant chemoprevention without clinical outcome evidence.

Abnormal autophagy and endoplasmic reticulum (ER) stress further contribute to OSF pathogenesis. Arecoline initially induces autophagy activation, evidenced by upregulation of LC3-II and Beclin-1 expression, whereas prolonged exposure leads to autophagic flux blockage, evidenced by p62 accumulation [115,116,117,118]. Peng et al. confirmed that autophagy inhibitors attenuate the degree of OSF, suggesting a dual role of autophagy in this condition [119]. Sustained ER stress induces apoptosis and promotes fibrosis through upregulation of the transcription factor CHOP [120].

Taken together, OSF-associated molecular alterations converge on a fibrosis-centered pathogenic program involving TGF-β/Smad activation, myofibroblast expansion, ECM remodeling, oxidative injury, autophagy/ER stress, and ncRNA-mediated regulation. Among these, ncRNAs should currently be interpreted as mechanistic candidates rather than clinically validated biomarkers, because most OSF-associated ncRNA findings remain insufficiently validated for routine risk stratification.

#### 4.1.2. Molecular Evolution Toward OSCC

The transformation of OSF to OSCC involves the sequential accumulation of molecular alterations. Genomic instability represents an early and critical event, manifested by increased micronucleus formation, chromosomal breakage, and dysregulation of DNA repair pathways [121]. A meta-analysis by Sarode et al. demonstrated that micronucleus frequency may serve as a predictive biomarker for malignant transformation risk in OSF [122]. Loss of heterozygosity (LOH) at chromosomal regions 9p21 (p16^INK4a^ locus), 3p (FHIT locus), and 17p13 (p53 locus) has been shown to increase progressively with lesion advancement [123]. In parallel, amplification of oncogenic loci, including 11q13 (Cyclin D1) and 7p12 (EGFR), contributes to enhanced proliferative signaling and malignant progression [124].

Emerging evidence from genomic studies indicates that mutational alterations accumulate during the early stages of oral carcinogenesis, including in potentially malignant disorders, supporting a stepwise model of malignant transformation [125]. Telomerase activation represents another key event in this process, with hTERT expression and telomerase activity progressively increasing from normal mucosa through OSF to OSCC [126,127,128]. Consistent with this observation, epigenetic regulation of the hTERT promoter, including increased methylation levels, has been implicated in telomerase reactivation during oral carcinogenesis [129]. Such reactivation enables replicative immortality, a hallmark of cancer, and contributes to the malignant transformation of OSF. Furthermore, elevated hTERT expression in OPMDs has been associated with an increased risk of malignant progression [130].

Oncogene activation and anti-oncogene inactivation synergistically drive OSF malignancy. The expression and activation levels of EGFR and its downstream Ras-MAPK and PI3K-AKT pathway components increase with lesion progression [131]. p53 mutation frequency rises from 10–20% in early-stage lesions to 40–50% in severe epithelial dysplasia and OSCC [132]. Tumor suppressor genes such as p16^INK4a^ and RUNX3 may be inactivated through promoter methylation and histone modification [133]. Reprogramming of the ncRNA regulatory network also plays an important role in OSF malignancy [134]. EMT activation is a characteristic event: with lesion progression, E-cadherin expression is downregulated while Vimentin and N-cadherin are upregulated, and EMT transcription factors such as Snail and Twist are increased [135]. This EMT process not only enhances migratory and invasive ability but also confers stem cell properties [136].

Microenvironmental remodeling further supports OSF malignant evolution. Shetty et al. demonstrated that altered EMT marker expression during microenvironmental remodeling creates favorable conditions for OSF malignancy [57]. Increased tissue stiffness directly affects epithelial cell proliferation and differentiation through integrin-FAK-Src and YAP/TAZ pathways [137,138,139,140]. Myofibroblasts secrete growth factors such as HGF and EGF, which promote epithelial cell proliferation and survival [141]. Altered vascular and lymphatic density, changes in inflammatory cell infiltration, and ECM remodeling collectively provide the basis for cancer cell growth and invasion [142].

Recent spatial transcriptome analyses have revealed dynamic changes characterizing the OSF malignant microenvironment [143]. The establishment of immune escape mechanisms represents a late event: epithelial cells evade immune surveillance by downregulating MHC-I expression and upregulating immune checkpoint molecules such as PD-L1 [144,145]. Concurrently, increased infiltration of Tregs and M2-type macrophages creates an immunosuppressive microenvironment [146,147]. Single-cell sequencing analysis showed that the inflammatory microenvironment shifted from pro-inflammatory to immunosuppressive during OSF malignancy [148]. Nevertheless, these high-resolution studies remain largely exploratory and require validation in larger, longitudinal cohorts. Their greatest current value is to identify candidate cellular interactions and molecular programs that may explain why only a subset of OSF lesions undergo malignant transformation.

In summary, OSF provides a strong example of exposure-driven, fibrosis-associated carcinogenesis. Its pathogenesis is supported by a coherent axis involving areca nut exposure, oxidative stress, TGF-β/Smad activation, ECM remodeling, tissue stiffness, and epithelial dysregulation. However, the relative contribution of each mechanism to malignant transformation remains difficult to quantify, particularly in patients with combined tobacco, alcohol, and areca nut exposure. Therefore, OSF risk assessment should integrate exposure history, clinical severity, fibrosis progression, epithelial dysplasia, and molecular markers rather than relying on any single pathway or biomarker.

### 4.2. Oral Lichen Planus (OLP)

#### 4.2.1. Immunopathological Mechanisms

OLP is a chronic inflammatory disease with a malignancy rate of approximately 1% [149]. In contrast to the fibrotic features of OSF, the core pathomechanism of OLP is a T cell-mediated autoimmune response targeting basal keratinocytes and the basement membrane zone [150]. CD8^+^ cytotoxic T lymphocyte (CTL)-mediated apoptosis of basal keratinocytes underlies the histopathology of OLP: activated CD8^+^ T cells recognize specific antigens on the surface of basal keratinocytes and induce apoptosis by releasing perforin, granzyme B, and FasL [151]. Sugerman et al. demonstrated that in OLP lesions, CD8^+^ T cells come into direct contact with apoptotic keratinocytes, resulting in the formation of characteristic “saw-tooth” rete ridge changes [152]. Recent single-cell analyses have revealed the heterogeneity of CD8^+^ T cell subpopulations and their distinct functional states in OLP [153].

An imbalance of T helper cell subpopulations plays a key role in OLP pathogenesis. The proportions of Th1 and Th17 cells are significantly increased in OLP lesions, while T helper 2 (Th2) cells are relatively decreased [154]. IFN-γ produced by Th1 cells activates macrophages and CTLs, enhancing cellular immune responses; IL-17 produced by Th17 cells recruits neutrophils and promotes the release of additional inflammatory factors [155]. Wang et al. demonstrated that an imbalance in the Th17/Treg ratio is closely related to the clinical manifestations and severity of OLP [156]. Abnormal chemokine networks are a key mechanism in the pathogenesis of OLP. CXCL9, CXCL10, CXCL11, and CCL5 expression is significantly upregulated in OLP lesions, selectively recruiting Th1 and CD8^+^ T cells to the lesion site through CXCR3 and CCR5 receptors [157]. The expression of ICAM-1 and VCAM-1 on endothelial cells is increased, promoting T cell extravasation and tissue infiltration [158]. Danielsson et al. demonstrated through transcriptome analysis that specific chemokine expression patterns can differentiate OLP subtypes and predict prognosis [159].

Impaired mucosal barrier function is an early event in OLP pathogenesis. The expression of oral mucosal tight junction proteins (claudin-1, occludin, and ZO-1) is downregulated in patients with OLP, leading to increased epithelial permeability and disruption of barrier function [160]. Barrier defects increase susceptibility to invasion by microorganisms and exogenous antigens, while promoting self-antigen exposure and triggering autoimmune responses [161].

NcRNAs may also participate in immune-mediated epithelial injury in OLP. Emerging evidence suggests that microRNAs (miRNAs), long non-coding RNAs (lncRNAs), and circular RNAs (circRNAs) can modulate keratinocyte apoptosis, T cell function, regulatory immune pathways, NF-κB signaling, and inflammatory persistence [162,163,164]. However, OLP-associated ncRNA studies remain limited and heterogeneous; therefore, these molecules should currently be regarded as exploratory mechanistic candidates rather than validated predictors of malignant transformation.

Importantly, OLP should not be framed as a uniformly high-risk premalignant condition. Its malignant potential remains more controversial than that of leukoplakia, erythroplakia, or OSF because diagnostic criteria, inclusion of oral lichenoid lesions, baseline epithelial dysplasia, and follow-up duration vary substantially across studies [11].

#### 4.2.2. Risk of Malignancy and Molecular Markers of OLP

Although the absolute malignant transformation risk of OLP is relatively low, long-term follow-up remains necessary because transformation may occur years after diagnosis, particularly in erosive or atrophic lesions and in patients with additional carcinogenic exposures [149]. At the molecular level, sustained activation of the STAT3 and NF-κB pathways, upregulation of COX-2 expression, and progressive epigenetic silencing of tumor suppressor genes may drive the malignant evolution of selected OLP cases [43,62]. The key pathogenic concept is not that all OLP lesions are premalignant in the same way, but that chronic immune-mediated epithelial injury may create repeated cycles of apoptosis, regeneration, DNA damage, and clonal selection.

Unlike other OPMDs, OLP malignancy is closely linked to its unique immunological milieu: the persistence of CD8^+^ T cell-mediated epithelial injury generates chronic DNA damage, and the progressive shift from a Th1/Th17-dominated response toward an immunosuppressive Th17/Treg imbalance creates conditions permissive for tumor escape [154,155]. Spatial transcriptome analysis has revealed that during OLP malignancy, immune cell subsets shift from pro-inflammatory to immunosuppressive phenotypes, with increased infiltration of CD163^+^ macrophages and FOXP3^+^ regulatory T cells [153]. Genomic instability markers, including micronucleus formation rate and LOH in chromosomal regions 9p21, 3p, and 17p13, are significantly elevated in OLP tissues that subsequently undergo malignant transformation and may serve as an important basis for malignant risk stratification [122,123]. Altered DNA methylation, with hypermethylation of the promoter regions of genes such as p16, E-cadherin, and DAPK, is correlated with malignancy risk [133]. However, no single molecular marker is currently sufficient to define malignant risk in OLP. A multiparameter assessment combining clinical subtype, lesion persistence, erosion or ulceration, histopathological findings, dysplasia status, immune cell infiltration pattern, and molecular alterations is more appropriate. This critical distinction is important because overestimating OLP risk may lead to unnecessary anxiety or overtreatment, whereas underestimating persistent erosive disease may delay recognition of malignant change.

### 4.3. Oral Leukoplakia (OLK)

#### 4.3.1. Clinical and Molecular Subtypes

OLK is the most common oral precancerous lesion and represents a clinically visible epithelial alteration with variable malignant potential [165]. Clinically, it is classified into homogeneous and non-homogeneous types, with the latter carrying a higher risk of malignant transformation [166]. Histologically, OLK is graded as no epithelial dysplasia, mild, moderate, or severe epithelial dysplasia; the degree of dysplasia correlates with malignancy risk but does not fully capture lesion behavior, field cancerization, or molecular progression [167].

Based on molecular features, OLK can be classified into high-proliferative (high expression of Ki-67, PCNA, Cyclin D1) and low-proliferative subtypes [168]; high-oxidative-stress (elevated 8-OHdG, MDA) and low-oxidative-stress subtypes [169]; stem cell-activated (high expression of CD44, CD133, ALDH1) and non-stem cell-activated subtypes [170]; and inflammation-driven (NF-κB and STAT3 activation) and non-inflammation-driven subtypes [171]. Molecular pathway typing based on EGFR/MAPK, PI3K/AKT, and Wnt/β-catenin activity reflects specific signaling pathway abnormalities and determines biological behavior and therapeutic sensitivity [172,173,174,175]. Molecular subtyping therefore provides the basis for accurate malignant risk assessment and individualized treatment strategies [176]. These molecular categories may help explain why clinically similar leukoplakic lesions behave differently. However, they remain more useful as research frameworks than as routine clinical classification systems. At present, clinical morphology, anatomical site, dysplasia grade, recurrence, and validated molecular markers remain more actionable for risk assessment than broad molecular subtype labels. Therefore, molecular subtyping should be presented as a promising research direction rather than as an established basis for individualized treatment.

#### 4.3.2. Pathways of Malignant Transformation

The malignant transformation of OLK to OSCC is a multistep, progressive process characterized by the sequential accumulation of genetic and epigenetic alterations [177]. Chromosomal abnormalities, particularly deletions at 9p21, 3p, and 17p13, represent the earliest detectable genetic alterations [153]. Abnormalities in cell cycle regulation manifest as p16 inactivation, Cyclin D1 upregulation, p53 mutation, and p27 downregulation, collectively leading to loss of cell cycle checkpoint function [178]. Altered telomere biology, including telomerase activation, upregulation of hTERT expression, and hTERT promoter mutations (C228T, C250T), contributes to replicative immortality [179]. Multiple cancer-related signaling pathways, including EGFR–Ras–MAPK, PI3K–AKT–mTOR, Wnt–β-catenin, are progressively and abnormally activated [180].

Epigenetic alterations encompass localized genomic hypermethylation, global hypomethylation, histone modification changes, and altered microRNA (miRNA) expression profiles [181]. In OLK, ncRNAs have been more directly investigated as candidate biomarkers of malignant transformation. Several miRNAs, including miR-21, miR-345, miR-181b, and miR-31, have been proposed as markers associated with leukoplakia progression [182]. Among them, miR-31 has relatively stronger longitudinal support, with increased expression associated with increased OPMDs progression risk [183]. CircHLA-C has also been reported as an upregulated circRNA and candidate biomarker in oral leukoplakia [184]. However, most ncRNA signatures in OLK remain insufficiently standardized across cohorts, sample types, and detection platforms.

Stem cell abnormalities are characterized by a shift from asymmetric to symmetric division, creating an “expanded stem cell reservoir” that fuels tumor growth [185]. Microenvironmental changes include the conversion of fibroblasts to CAFs, increased vascular and lymphatic vessel density, matrix remodeling, and altered immune cell infiltration [186]. Metabolic reprogramming, manifested by the Warburg effect, altered lipid metabolism, and abnormal amino acid metabolism, further supports malignant transformation [187]. Thus, OLK transformation should not be viewed as a purely epithelial genetic event. It reflects coordinated changes in epithelial clones, stromal cells, immune populations, vascular structures, and field mucosa.

Recent single-cell and spatial transcriptomic studies have provided important new insights into OLK malignancy. Sun et al. performed a comprehensive analysis of paired normal oral mucosa, OLK, and early OSCC samples and identified a set of “malignancy-associated” expression markers—including CTSC, FADD, ITGB4, LGALS1, LY6K, PMEPA1, TFAP2A, and TGFβI—that were progressively upregulated during malignant transformation [153]. TFAP2A, as a key transcription factor in the FRA pathway, increased in a stepwise manner from normal tissue through OLK to OSCC, and its knockdown significantly inhibited the proliferative capacity of OLK-like organoids. Furthermore, enrichment of a specific monocyte subpopulation (Mono_INHBA) in the OLK microenvironment and its frequent interactions with exhausted CD8^+^ T cells, CAFs, and epithelial cells collectively promote tumor microenvironment remodeling and immunosuppression. The spatial switching pattern of VEGF signaling between OLK and OSCC was also reported, with VEGF signaling enriched in the epithelial layer at the OLK stage redistributing throughout the full epithelial thickness by the OSCC stage, suggesting a critical role in basal epithelial cell proliferation.

Although these findings provide important mechanistic insight, they should be interpreted cautiously because spatial and single-cell datasets are often limited by sample size, lesion heterogeneity, and cross-sectional design. Their immediate value lies in hypothesis generation and biomarker discovery, rather than direct clinical decision-making.

In summary, OLK represents a clinically useful but biologically heterogeneous category. Histological dysplasia remains central to risk assessment, but it is not sufficient alone. Integration of clinical features, lesion site, recurrence pattern, exposure history, LOH status, epithelial proliferation markers, and emerging molecular data may improve risk stratification. However, clinical implementation requires prospective validation and demonstration that molecular testing changes management or improves outcomes.

### 4.4. Epigenetic and Post-Transcriptional Regulatory Alterations in OPMD Progression

Beyond genetic mutations and chromosomal instability, OPMD progression is also shaped by gene-regulatory alterations, including DNA methylation, histone modifications, chromatin remodeling, and ncRNA dysregulation [18,188,189]. These mechanisms can alter tumor suppressor gene expression, epithelial differentiation, inflammatory signaling, fibrosis, immune regulation, and field cancerization. Importantly, they may provide a molecular bridge between persistent inflammatory exposure and durable transcriptional reprogramming of oral epithelial and stromal cells.

DNA methylation is one of the most frequently investigated epigenetic alterations in oral premalignant progression. Promoter hypermethylation of tumor suppressor genes, including p16, E-cadherin, DAPK, and other cell cycle or adhesion-related genes, has been associated with epithelial dysregulation and increased malignant potential [188,190]. However, methylation markers should be interpreted cautiously because their clinical value depends on lesion subtype, sampling site, assay method, and integration with histopathological and clinical risk factors. At present, most methylation-based markers remain adjunctive rather than standalone predictors of malignant transformation [188,190].

Histone modifications and chromatin remodeling may further regulate inflammation-associated epithelial reprogramming. Altered histone acetylation, methylation, and related chromatin states can influence genes involved in proliferation, apoptosis resistance, EMT, immune signaling, and stromal interaction [191]. Emerging metabolism-linked modifications, such as histone lactylation, also suggest that inflammatory and metabolic changes in the oral microenvironment may affect gene expression during carcinogenesis; however, current evidence for lactylation is mainly derived from OSCC or broader head and neck cancer contexts rather than prospectively validated OPMD cohorts [192]. Therefore, compared with DNA methylation, histone-based biomarkers and therapeutic strategies remain less clinically developed in OPMDs, and much of the current evidence is preclinical or extrapolated from OSCC models.

NcRNAs provide an additional regulatory layer beyond classical epigenetic mechanisms. MiRNAs, lncRNAs, and circRNAs regulate gene expression through post-transcriptional repression, chromatin-associated regulation, molecular scaffolding, competing endogenous RNA activity, and miRNA-sponging mechanisms [189,193]. Disease-specific evidence suggests that ncRNAs may participate in different aspects of OPMD biology: lncRNAs may regulate TGF-β/Smad signaling and ECM remodeling in OSF [119,120]; ncRNAs may modulate keratinocyte apoptosis and immune regulation in OLP [162,163,164]; and miRNAs or circRNAs, such as miR-31 and circHLA-C, have been investigated as candidate biomarkers of OLK progression [182,183,184].

Despite their mechanistic relevance, epigenetic and ncRNA alterations are not yet ready for routine clinical decision-making in most OPMDs. Their translation is limited by lesion heterogeneity, variable sampling methods, inconsistent detection platforms, lack of standardized cutoffs, and insufficient prospective validation. Therefore, DNA methylation, histone modifications, and ncRNAs should currently be viewed as complementary tools for mechanistic insight, biomarker discovery, and future risk model refinement rather than as independent clinical determinants or established therapeutic targets.

### 4.5. Integrative Molecular Progression from OPMDs to OSCC

Comparing OPMDs and OSCC at the molecular level reveals key differential expression patterns. In epithelial cells, certain genes, including CDH1 and SPRR3, are upregulated at the OPMD stage but subsequently downregulated in OSCC, suggesting a protective role in early stages that is suppressed during tumor progression. In the stromal compartment, Mono_INHBA monocytes are enriched in OLK but decrease in OSCC; in contrast, Macro_APOE and Macro_NRG1 macrophage subpopulations are significantly increased in OSCC [194]. This dynamic shift reflects the transition from a pro-inflammatory to an immunosuppressive microenvironment. At the genomic level, LOH frequency at 9p21, 3p, and 17p13 increases progressively from OPMDs to OSCC; p53 mutations are less frequent in early-stage OPMDs but increase with severe epithelial dysplasia and OSCC [132]. mTORC1 and FRA pathway activity increase in a stepwise fashion from OPMDs to OSCC [153]. Epigenetically, p16 promoter methylation increases during progression, reflecting cumulative epigenetic disruption [133].

These molecular differences should not be interpreted as immediately actionable therapeutic targets in all OPMDs. Rather, they provide a framework for understanding disease progression, identifying candidate biomarkers, and refining risk stratification, while emphasizing that OSF, OLP, and OLK have both disease-specific mechanisms and shared malignant programs (Table 1).

## 5. Prevention and Risk-Adapted Intervention Strategies in Inflammation-Driven Oral Carcinogenesis

Because OPMDs represent clinically recognizable and potentially interceptable stages within inflammation-driven oral carcinogenesis, prevention and intervention strategies should be organized around biological risk, intervention window, and safety profile rather than the mere availability of therapeutic options. Effective prevention requires identifying which lesions are biologically likely to progress, which interventions can realistically modify that risk, and which strategies are sufficiently safe for long-term use in a premalignant population. This distinction is critical because most OPMDs do not inevitably progress to OSCC, whereas overtreatment may cause unnecessary morbidity and undertreatment may miss the opportunity for cancer interception. Therefore, risk-adapted decisions should integrate clinical phenotype, histopathological grade, molecular risk markers, etiological exposure, lesion behavior, patient adherence, and treatment-related toxicity [195,196,197,198].

A major limitation of the current OPMD intervention literature is that many studies use short-term clinical response, lesion size reduction, or histological improvement as endpoints rather than malignant transformation, cancer-free survival, recurrence, or quality of life [196]. This distinction directly affects interpretation. A treatment that reduces visible lesion burden may be useful for local disease control, but it does not necessarily prove prevention of malignant transformation, particularly in the presence of field cancerization. Conversely, a molecularly rational intervention may fail clinically if the selected target is not essential for lesion progression, if the biomarker is prognostic but not predictive, or if the toxicity profile is unacceptable for preventive use. Therefore, proposed interventions should be evaluated according to biological rationale, current evidence status, clinical applicability, and limitations rather than presented as equivalent therapeutic options.

Representative prevention and intervention strategies differ substantially in their biological rationale, evidence status, clinical applicability, and safety profile. Therefore, these strategies should be interpreted according to their position within the inflammation–OPMD–OSCC continuum, dominant target, current translational status, and major limitations (Table 2). This risk-adapted framework emphasizes that prevention in inflammation-driven oral carcinogenesis should be guided by biological risk and intervention window rather than by a treatment-centered catalog of available options. In exposure- or inflammation-dominant stages, exposure elimination, inflammatory control, patient education, and surveillance remain the foundation of prevention. For persistent, non-homogeneous, dysplastic, multifocal, recurrent, or molecularly high-risk lesions, management intensity should increase to include repeat biopsy, local control when indicated, molecular risk refinement, intensified surveillance, and consideration of clinical trial enrollment. Importantly, convergence on common pathways such as NF-κB, STAT3, COX-2/PGE_2_, EGFR, PI3K–AKT, or epigenetic/ncRNAs networks does not imply that pathway-targeted interventions are clinically validated or appropriate for routine preventive use.

### 5.1. Molecular Risk Assessment and Early Detection

Risk assessment and early detection based on molecular alterations have advanced considerably in OPMDs. Indicators of genomic instability—including LOH, microsatellite instability, and chromosomal copy number variations—have demonstrated predictive value for malignant transformation [199]. In particular, LOH at chromosomal regions 3p and 9p represents one of the most robustly validated risk markers in oral premalignant lesions [204]. However, molecular risk assessment should not be interpreted as a replacement for clinical examination or histopathological evaluation. Rather, its current role is to refine risk estimation in lesions with heterogeneous clinical behavior, borderline dysplasia, recurrence, multifocality, or discordance between clinical appearance and histological grade.

Additional alterations, including TP53 mutations, promoter hypermethylation of tumor suppressor genes such as p16, and EGFR overexpression, are associated with increased malignant potential [194]. Telomerase activation, including hTERT promoter alterations, has been proposed as an emerging biomarker, although its clinical utility remains to be fully established. Integrative models combining clinical, histological, and molecular parameters may improve risk stratification compared with single-parameter assessment [200]. Nevertheless, most candidate biomarkers remain insufficiently validated for routine standalone decision-making. Their clinical value depends on assay standardization, sampling site, lesion subtype, longitudinal reproducibility, and demonstration that biomarker-guided management improves clinically meaningful outcomes such as malignant transformation, recurrence, or cancer-free survival.

Noninvasive diagnostic approaches are also rapidly advancing. Liquid biopsy technologies—including circulating tumor DNA (ctDNA), exosomal miRNAs, and circulating tumor cells—offer promising tools for early detection and longitudinal monitoring [201]. Salivary biomarker panels, incorporating mutant DNA (e.g., TP53), methylated DNA (e.g., p16), and exosomal miRNAs, represent an attractive strategy for real-time surveillance. However, in the OPMD setting, these tools should currently be regarded as adjunctive rather than definitive diagnostic methods. Their major potential lies in monitoring field cancerization, identifying molecular changes during follow-up, and helping select sites for targeted biopsy, but large-scale prospective validation is still required before they can be integrated into routine clinical pathways.

Complementary imaging modalities such as narrow-band imaging, confocal microendoscopy, and Raman spectroscopy facilitate the identification of high-risk lesions and guide targeted biopsy [202]. In parallel, artificial intelligence and machine learning approaches are increasingly applied to integrate multi-dimensional datasets, further enhancing risk prediction models [203]. Despite this promise, AI-based tools require careful validation across institutions, imaging platforms, populations, and lesion subtypes. Algorithmic performance in retrospective datasets does not automatically translate into clinical reliability, and external validation is essential before these models can guide patient management.

The concept of field cancerization has important implications for surveillance design. Molecular alterations may extend beyond clinically visible lesions, necessitating monitoring strategies that encompass adjacent mucosa. Therefore, early detection should be understood as a longitudinal and field-oriented process rather than a single-lesion diagnostic event. Collectively, these advances provide the foundation for risk-adapted and stage-specific intervention, in which clinical phenotype, histological grade, molecular risk markers, field effects, and patient exposure history are integrated to guide surveillance intensity, biopsy strategy, and selection of local or investigational interventions.

### 5.2. Anti-Inflammatory, Antioxidant, Phytochemical, and Microenvironment-Directed Strategies

Chronic inflammation is a central biological driver of OPMD progression, making anti-inflammatory intervention conceptually attractive. Targeting the COX-2/PGE_2_ axis, NF-κB signaling, IL-6/STAT3 signaling, oxidative stress, microbial dysbiosis, and EMT-associated stromal remodeling could theoretically disrupt the pro-tumorigenic microenvironment that promotes genomic instability, epithelial proliferation, angiogenesis, immune escape, and early invasion-related processes [24,102,210,212]. However, this therapeutic area illustrates a recurring problem in OPMD prevention: mechanistic plausibility does not necessarily translate into clinical benefit. Therefore, anti-inflammatory and microenvironment-directed strategies should be interpreted according to evidence level, safety profile, and clinically meaningful endpoints rather than pathway rationale alone.

Selective COX-2 inhibitors such as celecoxib provide a clear example of this translational gap. COX-2 is frequently upregulated during oral carcinogenesis, and PGE_2_ promotes proliferation, angiogenesis, inflammation, and immune modulation. Observational studies have suggested a potential association between long-term NSAID use and reduced head and neck cancer incidence, and preclinical models support the biological relevance of COX-2/PGE_2_ inhibition [205]. Nevertheless, randomized clinical evidence has not established celecoxib as an effective preventive therapy for OPMDs. In a pilot randomized phase II trial of oral premalignant lesions, celecoxib at 100 or 200 mg twice daily was ineffective in controlling oral premalignant lesions [101]. In addition, cardiovascular toxicity concerns associated with long-term selective COX-2 inhibition limit its acceptability in a premalignant population [102]. Therefore, COX-2 inhibition should not be presented as routine chemoprevention, but rather as a biologically informative strategy that may guide future localized, short-term, or biomarker-selected interventions.

NF-κB and STAT3 are central inflammatory signaling nodes and remain attractive mechanistic targets. NF-κB inhibitors, including IKK inhibitors, ubiquitination-related inhibitors, and natural compounds such as curcumin and resveratrol, can suppress pro-inflammatory transcriptional programs in experimental settings [63]. STAT3 signaling can also be targeted through JAK inhibitors, SH2 domain-binding molecules, and oligonucleotide decoys [24]. However, direct pharmacological inhibition of NF-κB or STAT3 in OPMDs remains largely preclinical or early translational. Because both pathways participate in epithelial homeostasis, immune defense, tissue repair, and systemic inflammatory regulation, broad pathway blockade may carry unacceptable risk in a premalignant population. At present, these pathways are more useful for defining inflammation-dominant biology and identifying candidate trial targets than for routine clinical intervention.

Antioxidant therapy requires particular caution. Although ROS-mediated DNA damage is a key mechanism in inflammation-driven oral carcinogenesis, antioxidant supplementation has not consistently translated into cancer prevention in high-risk human populations. Agents such as vitamins C and E and N-acetylcysteine may reduce oxidative stress markers or support epithelial antioxidant defenses in selected contexts, but this does not prove durable prevention of malignant transformation. Landmark prevention trials in smokers challenged the assumption that antioxidant chemoprevention is uniformly beneficial. In the Beta-Carotene and Retinol Efficacy Trial (CARET), beta-carotene plus vitamin A had no preventive benefit and raised safety concerns, whereas the Alpha-Tocopherol, Beta-Carotene Cancer Prevention Study (ATBC) study found no reduction in lung cancer incidence with alpha-tocopherol or beta-carotene supplementation among male smokers [206,207]. These findings are clinically relevant to OPMDs populations because tobacco exposure is common among patients with oral premalignant lesions. Therefore, vitamins and antioxidant agents should be discussed as biologically plausible but clinically unproven interventions, and high-dose antioxidant supplementation should not be recommended as routine OPMD chemoprevention outside clinical trials.

Phytochemicals and natural compounds, including curcumin, green tea extract, resveratrol, and black raspberry preparations, have been investigated because they may modulate NF-κB, STAT3, COX-2, VEGF, oxidative stress, apoptosis, and epigenetic regulation [208,211,213]. Their relatively favorable toxicity profiles make them attractive for prevention, but their clinical evidence remains limited by small sample size, heterogeneous formulations, variable dosing, and reliance on short-term lesion response or biomarker modulation. For example, curcumin has shown potential benefit in OPMD management, and green tea extract has been tested in high-risk oral premalignant lesions, but neither can yet be considered proven to prevent malignant transformation [208,211]. Black raspberry gel is notable because topical application may modulate local biomarkers, including LOH-related changes, but this remains insufficient to establish long-term cancer-preventive efficacy [213]. Thus, phytochemicals should be framed as promising low-toxicity investigational agents rather than validated chemopreventive therapies.

Microbiome-targeted strategies, including probiotics and selective antimicrobial approaches, aim to restore oral microbial homeostasis and reduce inflammation-driven carcinogenic signaling [209]. This approach is biologically plausible because microbial dysbiosis can amplify Toll-like receptor signaling, cytokine production, acetaldehyde-related injury, and epithelial barrier dysfunction [210]. However, current evidence remains largely associative or preclinical, and it is difficult to determine whether microbial changes are causal drivers, modifiers, or consequences of OPMD progression. Therefore, microbiome-targeted strategies should currently be regarded as exploratory adjunctive approaches rather than established cancer-preventive interventions [210,212].

Anti-EMT and matrix-directed strategies, including TGF-β1 pathway modulation and MMP inhibition, may theoretically impede stromal remodeling and early invasion-related processes. These approaches are particularly relevant to fibrosis- or stroma-dominant lesions such as OSF, where TGF-β1 signaling, ECM deposition, tissue stiffness, and epithelial mechanotransduction are central to disease biology [223]. Nevertheless, systemic TGF-β1 or MMP inhibition may affect wound healing, immune regulation, and normal tissue remodeling, and clinical evidence supporting these strategies in OPMDs remains insufficient. Moreover, previous experience with broad MMP inhibitors in cancer therapy illustrates that strong preclinical rationale does not necessarily translate into clinical success [222]. Their most appropriate current role is as mechanistic trial concepts or localized intervention strategies in biologically selected high-risk lesions.

Finally, combination approaches targeting multiple inflammatory and microenvironmental pathways may ultimately prove more effective than single-agent strategies, particularly in inflammation-dominant or fibrosis-associated OPMDs. However, combination prevention is also more complex because additive toxicity, unclear target prioritization, and lack of validated response biomarkers can limit clinical feasibility. Future studies should therefore prioritize biomarker-defined patient selection, local delivery when possible, pharmacodynamic endpoints, and malignant transformation or recurrence as clinically meaningful outcomes.

### 5.3. Local Treatment and Photodynamic Therapy

Local treatment remains important for clinically persistent, symptomatic, non-homogeneous, or dysplastic OPMDs. Surgical excision and laser-based techniques can remove or reduce visible dysplastic epithelium and provide tissue for histopathological assessment. However, local treatment should be interpreted as lesion-directed management rather than complete biological prevention. Because molecular field cancerization may extend beyond the clinically visible lesion, recurrence, second primary lesions, and malignant transformation may still occur after apparently complete removal [196]. Therefore, local therapy is most appropriately used as part of a broader risk-adapted strategy that includes exposure control, histopathological assessment, and long-term surveillance.

Laser-based treatment may offer advantages such as reduced bleeding, precise ablation, good accessibility in the oral cavity, and acceptable functional outcomes. Nevertheless, the effect of laser treatment on malignant transformation remains uncertain. A systematic review and meta-analysis suggested that laser excision of oral leukoplakia may reduce recurrence in selected cases, but did not demonstrate a clear reduction in malignant transformation compared with conventional treatment [216]. This distinction is clinically important because reducing recurrence of a visible lesion is not equivalent to eliminating cancer risk. Patients treated with scalpel excision, laser excision, or laser ablation still require long-term surveillance, particularly when lesions are large, non-homogeneous, recurrent, multifocal, or histologically dysplastic.

Photodynamic therapy (PDT) represents a minimally invasive locoregional treatment that selectively targets dysplastic or neoplastic tissue. This approach involves administration of a photosensitizer followed by activation with a specific wavelength of light, resulting in the generation of cytotoxic reactive oxygen species [220]. The dual mechanism of PDT—direct induction of tumor cell apoptosis and disruption of lesion-associated microvasculature—makes it particularly suitable for localized OPMDs where preservation of surrounding normal tissue is essential. In addition, PDT may induce local inflammatory and immune responses, which may contribute to lesion regression, although the clinical relevance of this immunomodulatory effect in OPMDs remains incompletely established.

The most extensively studied photosensitizer in OPMDs is 5-aminolevulinic acid (5-ALA), which is metabolized intracellularly into the photoactive compound protoporphyrin IX. Topical 5-ALA-mediated PDT has emerged as an alternative minimally invasive therapeutic option for OLK, offering high selectivity and preservation of normal tissue architecture [217]. This tissue-sparing property is especially relevant for lesions located in functionally or esthetically sensitive oral sites, or for patients with multifocal superficial lesions in whom repeated conventional excision may cause morbidity.

Clinical evidence supporting PDT has accumulated. Some systematic reviews and meta-analyses have reported complete response rates of approximately 50–60% and high overall clinical response rates in selected OPMDs cohorts, particularly with 5-ALA-mediated protocols [214,215,219,221]. However, these findings mainly reflect short-term clinical lesion response rather than proven prevention of malignant transformation. Recurrence remains a concern, and the certainty of evidence is limited by small sample sizes, heterogeneous study designs, variable treatment protocols, and inconsistent follow-up duration.

Adverse effects, including transient pain and mucosal ulceration, are relatively common but generally manageable [218]. Compared with systemic pharmacological approaches, PDT has the advantage of local delivery and limited systemic toxicity. Nevertheless, recurrence remains a concern, and standardized protocols are still lacking. High-quality randomized controlled trials with adequate follow-up are needed to define optimal photosensitizer concentration, irradiation parameters, treatment intervals, retreatment strategies, and patient selection criteria. 

Taken together, PDT provides a mechanistically distinct and spatially targeted intervention that complements surgical and laser-based local therapies. Its strongest current role is as a tissue-preserving local disease-control strategy for selected superficial, multifocal, or surgically challenging OPMDs. However, PDT should not yet be presented as a proven malignant transformation-preventive intervention. Future studies should prioritize recurrence, malignant transformation, cancer-free survival, patient-reported outcomes, and treatment-related morbidity as clinically meaningful endpoints.

### 5.4. Molecular Targeted Therapy

Molecularly targeted therapies aim to inhibit key oncogenic signaling pathways that drive OPMD progression. These strategies are particularly relevant in high-risk lesions characterized by specific molecular alterations, including EGFR activation, PI3K–AKT–mTOR signaling, MAPK pathway activation, STAT3 signaling, VEGF-mediated angiogenesis, and other progression-associated molecular programs [224,230]. However, the preventive setting imposes a higher safety threshold than cancer treatment. Patients with premalignant lesions may remain stable for years, and systemic toxicity is difficult to justify unless the lesion has a clearly defined high risk of transformation and the intervention has a strong predictive biomarker. Therefore, molecularly targeted therapy should not be discussed as a generalized OPMDs treatment strategy, but rather as a biomarker-dependent investigational approach.

#### 5.4.1. EGFR-Targeted Strategies

EGFR overexpression and increased EGFR gene copy number have been reported in OSCC and high-risk oral premalignant lesions, providing a biological rationale for EGFR-targeted prevention [224]. Small-molecule tyrosine kinase inhibitors and monoclonal antibodies represent two major classes of EGFR-targeted agents. However, clinical evidence in OPMDs remains limited and should not be extrapolated directly from established OSCC or head and neck squamous cell carcinoma (HNSCC).

The Erlotinib Prevention of Oral Cancer (EPOC) trial is particularly important because it prospectively used LOH profiles to identify high-risk oral premalignant lesions. Contrary to the interpretation that LOH-positive high-risk lesions may benefit from EGFR inhibition, erlotinib did not significantly improve cancer-free survival in LOH-positive patients, including those with increased EGFR gene copy number [225]. Therefore, the key lesson from EPOC is not that EGFR inhibition benefits LOH-positive lesions, but that molecular risk stratification is feasible and that risk biomarkers do not automatically predict therapeutic response.

This distinction is central to precision prevention. A biomarker may be prognostic, meaning that it identifies lesions likely to progress, without being predictive, meaning that it identifies lesions likely to respond to a specific drug. LOH is valuable for risk stratification, but EPOC does not support erlotinib as an effective preventive therapy for LOH-positive oral premalignant lesions [225]. Future targeted prevention trials should therefore incorporate both risk enrichment biomarkers and drug-specific predictive biomarkers, along with pharmacodynamic endpoints, toxicity monitoring, and clinically meaningful outcomes such as malignant transformation, recurrence, and cancer-free survival.

Anti-EGFR monoclonal antibodies, most notably cetuximab, target the extracellular domain of EGFR and have demonstrated survival benefit in combination with radiotherapy for locoregionally advanced HNSCC [229]. However, this evidence comes from established invasive cancer, not from OPMDs. Therefore, cetuximab should not be presented as a therapeutic escalation option for high-risk OPMDs outside clinical trials. Its systemic toxicity profile, including acneiform rash, infusion reactions, and gastrointestinal adverse effects, further limits its acceptability in a premalignant population. At present, anti-EGFR approaches in OPMDs should be regarded as investigational and should require molecular selection, local or toxicity-sparing delivery strategies, and prospective clinical validation.

#### 5.4.2. PI3K–AKT–mTOR, MAPK, and Anti-Angiogenic Strategies

PI3K–AKT–mTOR and MAPK signaling regulate epithelial proliferation, survival, metabolism, apoptosis resistance, and stemness-associated phenotypes during oral carcinogenesis [226,230]. Activation of these pathways may occur during the transition from OPMDs to OSCC, making them attractive candidates for molecular intervention. However, pathway activation alone is insufficient to justify systemic targeted therapy in premalignant disease. Most evidence supporting PI3K–AKT–mTOR or MAPK pathway targeting in OPMDs remains preclinical, exploratory, or extrapolated from OSCC and HNSCC treatment studies. Such extrapolation should be made cautiously because pathway dependence in invasive cancer may not be identical to pathway dependence in the premalignant epithelium.

mTOR-related prevention strategies provide an example of both promise and limitation. A phase IIa trial in individuals with oral premalignant lesions explored metformin as a strategy to inhibit mTOR signaling and reported biological activity, supporting the feasibility of pathway modulation in selected OPLs [228]. Nevertheless, this does not establish mTOR-targeted therapy as routine OPMDs management. Future studies must determine which lesions are pathway-dependent, whether pharmacodynamic modulation is durable, and whether pathway inhibition reduces malignant transformation rather than only modifying short-term molecular markers.

Angiogenesis is also implicated in OPMD progression. VEGF expression and subepithelial vascularization have been reported to increase in oral leukoplakia, particularly in lesions with dysplastic changes, supporting a mechanistic link between angiogenesis and malignant progression [227]. In the OPMD setting, anti-VEGF or broader anti-angiogenic strategies are conceptually attractive because VEGF expression and angiogenesis-related programs appear to increase during progression from OPMDs to OSCC, including in spatial transcriptomic analyses [153]. However, clinical evidence supporting anti-angiogenic treatment in OPMDs remains insufficient. Moreover, systemic inhibition of angiogenesis may carry risks related to wound healing, vascular homeostasis, and tissue repair. Therefore, VEGF-related pathways are currently more useful as indicators of microenvironmental remodeling and potential trial targets than as established therapeutic targets in premalignant oral disease. Future studies may evaluate anti-angiogenic modulation as part of biomarker-defined or locally delivered multimodal strategies, but such approaches should remain investigational until clinical benefit and safety are demonstrated.

Overall, molecular targeted therapy in OPMDs should be framed as a future precision-prevention strategy rather than an established treatment category. Rational application would require molecularly defined high-risk lesions, evidence of pathway dependence, validated predictive biomarkers, acceptable toxicity, and demonstration of benefit on clinically meaningful endpoints such as malignant transformation, recurrence, or cancer-free survival. Without these conditions, targeted therapy risks becoming a mechanism-driven but clinically unsupported approach.

### 5.5. Immune Checkpoint Blockade and Immunomodulation

Immune dysregulation plays a pivotal role in OPMD progression, but the immune microenvironment of premalignant lesions differs from that of established OSCC [232]. In advanced or recurrent/metastatic HNSCC, immune checkpoint blockade has demonstrated clinical benefit, particularly through programmed cell death protein 1 (PD-1)/ programmed death-ligand 1 (PD-L1)-targeted strategies [234,235]. In OPMDs, however, immune alterations are more heterogeneous and may include chronic inflammatory infiltration, Th17/Treg imbalance, M2 macrophage accumulation, impaired antigen presentation, stromal remodeling, and progressive immune suppression [147,231]. Therefore, direct application of systemic anti-PD-1/PD-L1 antibodies to premalignant disease is not currently justified by available evidence.

In the OPMD setting, immune checkpoint biology should be interpreted as a marker of immune evolution rather than as an immediate indication for checkpoint blockade. Single-cell and spatial transcriptomic analyses of adjacent dysplastic/OLK lesions and early OSCC have revealed progressive immune microenvironmental remodeling during OSCC initiation, including enrichment of the immune-inhibitory Mono_INHBA monocyte subset, interactions with exhausted CD8^+^ T cells through CD274–PDCD1 and PVR/NECTIN2–TIGIT axes, macrophage remodeling, TNFRSF4^+^ Treg enrichment, and altered epithelial–immune–stromal communication [153]. These findings support the concept that checkpoint-associated immune escape may emerge during malignant progression, but they do not establish systemic checkpoint blockade as a preventive intervention for OPMDs. The key unresolved question is whether immune checkpoint-related signaling in premalignant lesions is a driver of progression, a marker of immune adaptation, or a consequence of broader inflammatory remodeling.

Compared with systemic immune checkpoint blockade, a more plausible near-term direction is local or pathway-specific immunomodulation [233]. STAT3 inhibition represents a mechanistically attractive strategy because STAT3 links inflammatory cytokine signaling with epithelial survival, immune suppression, angiogenesis, and checkpoint-associated regulation. Inhibiting STAT3 could theoretically restore CD8^+^ T cell function, reduce MDSC and Treg expansion, attenuate tumor-promoting cytokine loops, and decrease immune-suppressive signaling [24]. However, direct STAT3 inhibition in OPMDs remains largely preclinical or early translational. Because STAT3 also participates in normal epithelial repair, immune regulation, and tissue homeostasis, systemic inhibition may not be acceptable for routine prevention unless lesion selection, delivery route, and toxicity are carefully controlled [236,237]. Macrophage repolarization strategies, which aim to shift M2-like immunosuppressive macrophage programs toward more pro-inflammatory or anti-tumor phenotypes, represent another emerging approach. This concept may be particularly relevant to inflammation- or fibrosis-associated OPMDs, including OSF and OLP, where M2 macrophage accumulation and macrophage-associated stromal remodeling have been implicated in disease progression and malignant risk [147,148]. Nevertheless, macrophage-directed intervention remains investigational. The macrophage compartment is highly plastic and context-dependent, and broad manipulation of macrophage function may affect wound healing, microbial defense, and tissue repair. Therefore, macrophage repolarization should currently be viewed as a mechanistic trial concept rather than an established preventive strategy. TLR agonist-based immunostimulators and other local immune-modulating approaches may provide a more controlled way to reactivate innate and adaptive immune surveillance in selected lesions. Local delivery is conceptually attractive because it may enhance lesion-specific immune activation while reducing systemic toxicity [233]. However, the optimal immune target, dose, formulation, treatment interval, and patient selection criteria remain undefined. Moreover, excessive immune stimulation could theoretically worsen immune-mediated lesions such as OLP. Thus, locally delivered immunomodulation should be explored cautiously within well-designed clinical trials.

Overall, immunomodulation should be framed as a promising but immature area of OPMD prevention. Its future development should prioritize lesion-specific immune profiling, spatial immune mapping, local delivery strategies, toxicity monitoring, and biomarkers that can distinguish immune activation associated with lesion control from immune suppression associated with malignant progression. Until such evidence is available, systemic immune checkpoint blockade should remain outside routine OPMD management, and immunomodulatory approaches should be considered investigational.

### 5.6. Epigenetic and Post-Transcriptional Regulatory Strategies

Epigenetic alterations represent early and potentially reversible events in oral carcinogenesis, making them attractive targets for intervention. DNA methylation, histone modifications, chromatin remodeling, histone lactylation, and ncRNA dysregulation can regulate tumor suppressor gene expression, epithelial differentiation, inflammatory signaling, fibrosis, metabolism, immune regulation, and field cancerization [188,190,191,192,241]. However, reversibility at the molecular level does not automatically imply therapeutic feasibility. In the premalignant setting, intervention must meet a higher safety threshold than cancer treatment because many OPMDs may remain stable for years and systemic toxicity is difficult to justify without clear evidence of high transformation risk.

DNA methyltransferase (DNMT) inhibitors and histone deacetylase (HDAC) inhibitors can restore tumor suppressor gene expression and modulate oncogenic transcriptional programs in cancer models [192,241]. In OPMDs, however, their routine use is not clinically established. Although promoter methylation of tumor suppressor genes such as p16 has been associated with malignant transformation risk in oral epithelial dysplasia, methylation status is more appropriately viewed as a candidate risk marker than as a direct indication for systemic epigenetic therapy [188,190]. Systemic toxicity and broad off-target effects currently limit the application of conventional epigenetic drugs in premalignant oral disease, making localized delivery, topical formulations, or short-term pharmacodynamic trials more realistic than routine systemic epigenetic treatment [241,242].

Histone modifications and chromatin remodeling provide another layer of gene regulation during oral carcinogenesis. Altered histone acetylation and methylation can influence epithelial proliferation, apoptosis resistance, EMT, immune signaling, and stromal interaction [191,241]. Emerging evidence suggests that metabolism-linked histone modifications, such as histone lactylation, may connect metabolic reprogramming with tumor-promoting transcriptional programs in OSCC [192]. However, histone lactylation should be interpreted cautiously in the OPMDs context because current evidence is mainly derived from OSCC or broader oral cancer models rather than prospectively validated OPMDs cohorts. Thus, histone-based mechanisms are biologically important, but histone modification-targeted therapy remains clinically underdeveloped for OPMD management.

Natural compounds with epigenetic or transcriptional regulatory activity, including curcumin and green tea polyphenols, may offer lower-toxicity alternatives to conventional epigenetic drugs [208,211]. However, these agents are pleiotropic, and their effects are difficult to attribute to specific epigenetic mechanisms. Their reported benefits in OPMDs are generally based on short-term clinical response, biomarker modulation, or small clinical studies rather than durable prevention of malignant transformation. Therefore, natural epigenetic modulators should be framed as low-toxicity investigational agents rather than validated epigenetic chemopreventive therapies.

NcRNA-based approaches, including miRNA mimics, antagomirs, lncRNA inhibitors, antisense oligonucleotides, and RNA interference-based strategies, are also conceptually attractive because they could modulate multiple inflammatory and oncogenic pathways simultaneously [189,243]. However, RNA-based therapeutics face major translational challenges, including tissue-specific delivery, RNA stability, off-target effects, immune activation, dosing, and long-term safety. In OPMDs, the strongest current value of ncRNAs is more likely to lie in biomarker discovery, risk-model refinement, and longitudinal surveillance than in immediate therapeutic application.

Overall, epigenetic and post-transcriptional regulatory strategies should be regarded as biologically promising but clinically immature in OPMD prevention. Their future development will require lesion-specific molecular risk stratification, standardized assays, local or toxicity-sparing delivery approaches, pharmacodynamic endpoints, and demonstration of benefit on clinically meaningful outcomes such as recurrence, malignant transformation, or cancer-free survival.

### 5.7. Toward a Risk-Adapted Clinical Management Framework

Based on the current evidence, prevention and intervention in inflammation-driven oral carcinogenesis should be organized around biological risk rather than around the mere availability of therapeutic options. A practical framework should first define lesion risk using clinical phenotype, histopathological grade, anatomical site, lesion size, recurrence pattern, etiological exposure, and, when available, molecular markers such as LOH at 3p and/or 9p, DNA aneuploidy, p16 methylation, TP53 alteration, or other validated risk-associated biomarkers [5,195,197,198,204,238,239]. This approach avoids two common pitfalls: overtreating lesions with low biological risk and undertreating lesions with high-risk features but modest clinical appearance. Risk-adapted management should therefore integrate exposure control, biopsy strategy, local treatment, molecular surveillance, follow-up intensity, and patient-related factors [195,197,198].

Low-risk OPMDs may include homogeneous leukoplakia without epithelial dysplasia, small lesions at lower-risk anatomical sites, lesions without rapid clinical change, and patients without persistent high-risk exposures [195,197,198,239]. In these cases, management should prioritize elimination of etiological drivers, including tobacco, alcohol, areca nut, and chronic mechanical irritation; clinical photography; patient education; and periodic surveillance [195,197,198]. Biopsy remains necessary when lesions persist, enlarge, become non-homogeneous, ulcerate, or change symptomatically [195,198]. The key principle is that low-risk management should not mean absence of follow-up, because molecular field changes and patient exposure patterns may evolve over time [103].

Intermediate-risk OPMDs include persistent or recurrent leukoplakia, non-homogeneous lesions, moderate epithelial dysplasia, large lesions, lesions located on high-risk sites such as the lateral tongue or floor of mouth, and lesions occurring in patients with ongoing carcinogenic exposure [197,239]. These lesions require closer surveillance, repeat biopsy when clinical changes occur, and consideration of local therapy, including excision, laser-based treatment, or PDT, depending on lesion size, location, functional impact, and patient factors [195,196,197,198,216,218]. Molecular testing may be useful when available, particularly if histopathological findings are borderline or clinical behavior is discordant with dysplasia grade [204,238]. The aim in this group is to prevent delayed recognition of progression while avoiding unnecessary aggressive treatment.

High-risk OPMDs include erythroplakia, proliferative verrucous leukoplakia, severe epithelial dysplasia, carcinoma in situ, multifocal or recurrent non-homogeneous lesions, lesions with rapid clinical progression, and lesions harboring high-risk molecular alterations such as LOH at 3p and/or 9p [5,197,204,238,239,240]. These patients should undergo definitive local management when feasible, mapping biopsy for multifocal or clinically heterogeneous disease, intensified surveillance, and consideration of referral to centers capable of molecular risk assessment or clinical trial enrollment [195,197,198]. Importantly, high-risk management should not rely only on visible lesion removal. Because field cancerization may persist beyond the treated area, long-term surveillance of adjacent and distant oral mucosa remains necessary [103].

**Figure 4:**
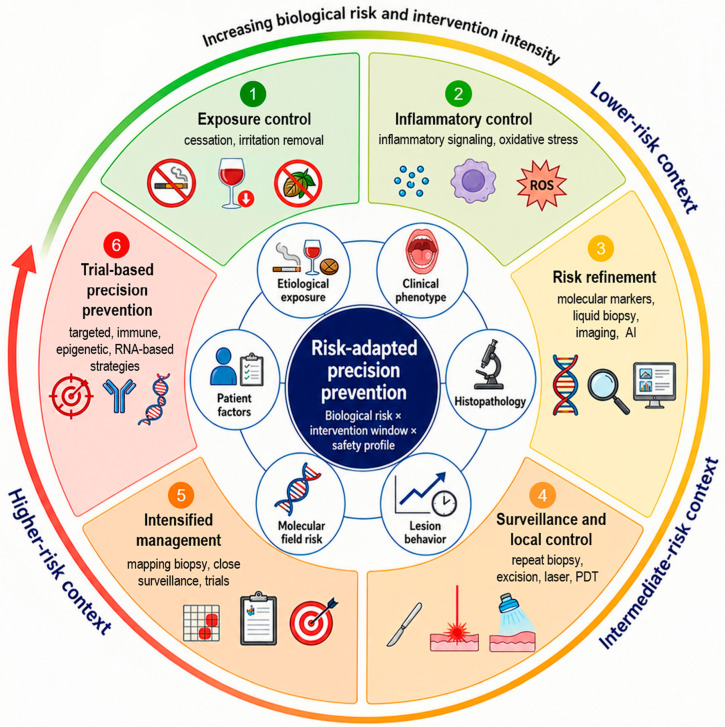
The risk-adapted prevention and intervention framework in inflammation-driven oral carcinogenesis. The figure illustrates a circular framework for organizing prevention and intervention strategies according to biological risk, intervention window, and safety profile. Risk assessment integrates etiological exposure, clinical phenotype, histopathological findings, lesion behavior, molecular field risk, and patient-related factors. Intervention windows progress from exposure control and inflammatory control to risk refinement, surveillance and local control, intensified management, and trial-based precision prevention. As biological risk increases, intervention intensity may also increase, ranging from cessation of carcinogenic exposures and surveillance to molecular testing, local treatment, intensified follow-up, and investigational precision-prevention strategies. This framework is intended as a conceptual model rather than a rigid clinical algorithm.

This framework should be interpreted as a flexible risk-adapted model rather than a rigid clinical guideline. OPMDs are biologically heterogeneous, and the optimal strategy depends on lesion subtype, patient risk factors, available diagnostic resources, treatment morbidity, patient adherence, and patient preferences [5,197]. The major challenge in OPMDs prevention is not the absence of candidate pathways, but the lack of validated strategies to identify which lesion should receive which intervention at which stage [196]. Future studies should therefore move beyond short-term lesion regression and prioritize malignant transformation, recurrence, cancer-free survival, molecular response, patient-reported outcomes, and treatment-related morbidity [196,216,218]. Overall, a mechanism-informed, evidence-informed, and risk-adapted framework is more clinically realistic than uniform chemoprevention or lesion-directed treatment alone (Figure 4).

## 6. Conclusions and Outlooks

OPMDs represent clinically recognizable and potentially interceptable stages within inflammation-driven oral carcinogenesis. Chronic inflammation links etiological exposures, epithelial injury, immune cell recruitment, stromal remodeling, oxidative DNA damage, and molecular field alterations to the stepwise development of OSCC. However, the etiological drivers of this process should not be interpreted as equivalent. Tobacco, alcohol, and areca nut/betel quid exposure have stronger causal and mechanistic support, whereas microbial dysbiosis, HPV infection, and immune-mediated epithelial injury appear to act in more context-dependent ways. This distinction is important because prevention should prioritize elimination of validated carcinogenic exposures while using clinicopathological and molecular features to refine risk in heterogeneous lesions.

At the mechanistic level, persistent activations of NF-κB, STAT3, COX-2/PGE_2_, oxidative stress responses, cytokine and chemokine networks, and epithelial–stromal interactions create a permissive microenvironment for clonal expansion, immune escape, angiogenesis, EMT, and invasion. In parallel, genomic instability, DNA methylation, histone modifications, histone lactylation, and ncRNA dysregulation may stabilize inflammation-induced transcriptional programs and contribute to field cancerization. These mechanisms provide important biological insight, but they should not be interpreted as immediately actionable therapeutic targets in all OPMDs. The key translational challenge is to distinguish molecular alterations that merely accompany lesion progression from those that predict risk, define pathway dependence, or guide intervention.

Clinically, OPMDs should be approached as a heterogeneous group of disorders rather than as a uniform premalignant entity. Lesion subtype, anatomical site, clinical morphology, histopathological grade, etiological exposure, recurrence pattern, field effects, and molecular markers such as LOH at 3p and/or 9p should be integrated to refine risk stratification. Emerging tools, including salivary and liquid biopsy biomarkers, optical imaging, spatial transcriptomics, and artificial intelligence-assisted models, may further improve risk prediction, but most require assay standardization, external validation, and demonstration that biomarker-guided management improves clinically meaningful outcomes.

From a therapeutic perspective, the current evidence supports a cautious and risk-adapted interpretation. Exposure elimination, biopsy-based diagnosis, appropriate local control, and long-term surveillance remain the foundation of management, whereas many mechanism-based interventions—including COX-2 inhibition, antioxidant supplementation, molecular targeted therapy, immune checkpoint blockade, epigenetic drugs, and ncRNA-based therapeutics—remain limited by inconsistent efficacy, toxicity concerns, delivery barriers, or insufficient OPMD-specific clinical validation. PDT and laser-based approaches may provide useful local disease control in selected lesions, but lesion regression or recurrence reduction should not be equated with proven prevention of malignant transformation.

Future research should therefore move beyond short-term lesion response and prioritize malignant transformation, recurrence, cancer-free survival, molecular response, patient-reported outcomes, and treatment-related morbidity. Longitudinal cohorts, biomarker-defined trials, local or toxicity-sparing delivery strategies, and integrated multi-omics models will be essential for determining which lesions require intensified intervention and which strategies are sufficiently safe for preventive use. Overall, a mechanism-informed, evidence-informed, and risk-adapted framework is more clinically realistic than uniform chemoprevention or lesion-directed treatment alone. Such an approach may help transform inflammation-driven oral carcinogenesis from a late-detected malignant process into a preventable and interceptable disease continuum.

## Figures and Tables

**Table 1:** Disease-specific inflammatory and molecular mechanisms and translational implications in major OPMDs.

OPMDs Subtype	Dominant Pathological Context	Major Inflammatory and Molecular Mechanisms	Malignant Transformation-Associated Programs	Risk Stratification and Translational Implications
Oral submucous fibrosis (OSF)	Areca nut/betel quid exposure; chronic fibrosis; mucosal stiffness; restricted mouth opening	TGF-β1/Smad activation; fibroblast-to-myofibroblast transition; ECM deposition; MMP/TIMP imbalance; lysyl oxidase-mediated collagen cross-linking; ROS/RNS-mediated injury; autophagy and ER stress; ncRNA-mediated profibrotic regulation	Genomic instability; micronucleus formation; LOH at 9p21, 3p, and 17p13; telomerase activation; EMT; mechanotransduction through integrin–FAK–Src and YAP/TAZ; immune suppression; OSF-to-OSCC microenvironmental remodeling	Risk assessment should integrate exposure history, fibrosis severity, mouth-opening limitation, epithelial dysplasia, oxidative/genomic instability markers, and molecular alterations. Areca nut cessation, fibrosis control, and long-term surveillance are central preventive priorities.
Oral lichen planus (OLP)	Chronic immune-mediated epithelial injury; reticular, atrophic, or erosive lesions; basal keratinocyte damage	CD8^+^ T cell cytotoxicity; Fas/FasL and perforin/granzyme-mediated apoptosis; Th1/Th17-skewed inflammation; Th17/Treg imbalance; IFN-γ, TNF-α, IL-17, and chemokine networks; barrier dysfunction; NF-κB/STAT3 activation; exploratory ncRNA-mediated immune regulation	Repeated epithelial apoptosis–regeneration cycles; chronic inflammatory and oxidative stress; immune microenvironmental remodeling; epigenetic silencing of tumor suppressor genes; genomic instability in selected high-risk lesions	Risk evaluation should emphasize erosive or ulcerative subtype, lesion persistence, epithelial dysplasia, coexisting carcinogenic exposure, immune cell infiltration pattern, and longitudinal clinical change. Long-term surveillance remains necessary.
Oral leukoplakia (OLK)	Clinically visible white plaque; homogeneous or non-homogeneous morphology; variable epithelial dysplasia; field cancerization	EGFR–RAS–MAPK signaling; PI3K–AKT–mTOR activation; Wnt/β-catenin signaling; NF-κB/STAT3 activation; oxidative stress; altered keratinocyte proliferation and apoptosis; stemness-associated markers; metabolic reprogramming; epigenetic and ncRNA dysregulation	LOH at 3p, 9p21, and 17p13; p16 inactivation; cyclin D1 upregulation; TP53 alteration; hTERT activation; EMT; CAF activation; vascular/lymphatic remodeling; macrophage and monocyte remodeling; OLK-to-OSCC spatial transcriptomic progression	Risk evaluation should emphasize erosive or ulcerative subtype, lesion persistence, epithelial dysplasia, coexisting carcinogenic exposure, immune cell infiltration pattern, and longitudinal clinical change. Long-term surveillance remains necessary.
Shared malignant programs across OPMDs	Persistent inflammatory or carcinogenic exposure; lesion-specific epithelial–stromal–immune interactions	Cytokine and chemokine amplification; ROS/RNS accumulation; NF-κB, STAT3, COX-2/PGE_2_, TGF-β, EGFR/MAPK, PI3K–AKT–mTOR, Wnt/β-catenin, and YAP/TAZ signaling; DNA methylation; histone modification; ncRNA dysregulation	Field cancerization; clonal expansion; genomic instability; epithelial dysplasia; EMT; angiogenesis; CAF activation; macrophage polarization; immune escape; basement membrane disruption and invasion	Risk evaluation should emphasize erosive or ulcerative subtype, lesion persistence, epithelial dysplasia, coexisting carcinogenic exposure, immune cell infiltration pattern, and longitudinal clinical change. Long-term surveillance remains necessary.

**Table 2:** Risk-adapted intervention windows along the inflammation–OPMD–OSCC continuum.

Intervention Window	Dominant Biological or Clinical Problem	Representative Strategies	Current Evidence/ Translational Status	Critical Appraisal and Clinical Limitations
Etiological exposure and chronic mucosal injury	Persistent carcinogenic exposure and mucosal irritation promote epithelial injury, oxidative stress, inflammatory signaling, and field cancerization pressure.	Tobacco cessation; alcohol reduction; areca nut/betel quid cessation; removal of chronic mechanical irritation; patient education; periodontal and mucosal inflammatory control.	Foundational prevention principle supported by epidemiological and clinical evidence.	Essential and low-risk, but exposure elimination alone may not reverse established molecular field changes. Persistent, recurrent, non-homogeneous, or dysplastic lesions still require biopsy-based assessment and long-term surveillance [103,195,197,198].
Early molecular field alteration and risk detection	Genomic instability, clonal expansion, epigenetic alteration, field cancerization, and molecular heterogeneity may precede invasive carcinoma.	Clinical examination; histopathological grading; LOH analysis; DNA aneuploidy; TP53 alteration; p16 methylation; EGFR overexpression; salivary/liquid biopsy biomarkers; optical imaging; AI-assisted risk prediction.	LOH at 3p/9p has relatively stronger validation; most other biomarkers, liquid biopsy approaches, imaging adjuncts, and AI models remain adjunctive or investigational.	Molecular tools should refine, not replace, clinical examination and histopathology. Clinical adoption requires assay standardization, external validation, and evidence that biomarker-guided management improves outcomes [194,199,200,201,202,203,204].
Inflammation-dominant remodeling	COX-2/PGE_2_ activation, NF-κB and IL-6/STAT3 signaling, oxidative injury, microbial dysbiosis, and cytokine amplification create a pro-tumorigenic inflammatory microenvironment.	COX-2/PGE_2_ inhibition; NSAID-related approaches; NF-κB or STAT3 pathway modulation; antioxidant agents; microbiome-targeted strategies; selected phytochemicals.	Strong mechanistic rationale, but clinical translation remains limited, inconsistent, or investigational.	Celecoxib did not establish effective OPMD chemoprevention, and long-term selective COX-2 inhibition raises cardiovascular safety concerns. CARET and ATBC caution against high-dose antioxidant prevention. Microbiome-targeted strategies remain exploratory [24,63,101,102,205,206,207,208,209,210,211,212,213].
Visible, persistent, or dysplastic OPMDs requiring local control	Clinically persistent, recurrent, non-homogeneous, symptomatic, or dysplastic lesions require lesion-directed management, but surrounding field changes may persist.	Scalpel excision; laser excision or ablation; PDT; repeat biopsy; site-specific local management.	Local therapies are clinically used for lesion control; PDT and laser treatment have supportive clinical evidence, but long-term transformation endpoints remain limited.	Lesion regression or recurrence reduction should not be equated with cancer prevention. Laser treatment may reduce recurrence in selected cases, but malignant transformation benefit remains uncertain. PDT is tissue-preserving, but protocols and follow-up endpoints are heterogeneous [196,214,215,216,217,218,219,220,221].
Fibrosis- and stroma-associated progression	ECM deposition, stromal stiffness, TGF-β1 signaling, EMT, MMP activity, mechanotransduction, and epithelial–stromal crosstalk may promote malignant susceptibility, especially in fibrosis-dominant contexts such as OSF.	Areca nut cessation; fibrosis monitoring; management of functional limitation; investigational TGF-β1 pathway modulation; MMP-related or matrix-directed approaches.	Exposure elimination and surveillance are clinically relevant; anti-fibrotic and matrix-directed strategies remain mainly mechanistic or investigational in OPMDs.	Systemic TGF-β1 or MMP inhibition may affect wound healing, immune regulation, and tissue repair. Previous experience with broad MMP inhibitors illustrates that strong preclinical rationale does not necessarily translate into clinical success [195,197,198,222,223].
Oncogenic pathway activation during high-risk transition	EGFR, PI3K–AKT–mTOR, MAPK, VEGF, angiogenesis-related programs, and metabolic reprogramming may support epithelial proliferation, survival, and progression toward OSCC.	EGFR inhibition in trial settings; metformin/mTOR modulation; PI3K or MAPK pathway targeting; VEGF/anti-angiogenic trial concepts.	Biomarker-driven and early-phase studies exist, but routine molecular targeted therapy is not established for OPMDs.	EPOC supports feasibility of molecular risk stratification but not erlotinib efficacy. LOH is prognostic but not necessarily predictive of EGFR inhibitor response. Pathway activation alone does not justify systemic targeted therapy [153,204,224,225,226,227,228,229,230].
Immune escape and immune microenvironment remodeling	T cell exhaustion, checkpoint-associated signaling, Th17/Treg imbalance, M2 macrophage polarization, cytokine loops, and impaired immune surveillance may emerge during progression.	Immune profiling; local or pathway-specific immunomodulation; STAT3 inhibition; macrophage repolarization; TLR agonist-based local immunostimulation.	Checkpoint blockade is established in advanced HNSCC, but preventive use in OPMDs remains investigational. Local or pathway-specific immunomodulation is early-stage.	Systemic immune checkpoint blockade is not justified for routine premalignant disease. Immunomodulation requires lesion-specific immune profiling, local delivery strategies, toxicity monitoring, and validated immune response biomarkers [24,147,148,153,231,232,233,234,235,236,237].
Epigenetic and post-transcriptional dysregulation	DNA methylation, histone modification, histone lactylation, chromatin remodeling, and ncRNA networks may stabilize inflammation-induced transcriptional programs and molecular field changes.	DNMT inhibitors; HDAC inhibitors; topical/local epigenetic modulation; histone lactylation-related targets; miRNA mimics; antagomirs; lncRNA inhibitors; antisense oligonucleotides.	Biologically promising but clinically immature; mainly biomarker, preclinical, or OSCC-extrapolated evidence.	Reversibility does not imply therapeutic feasibility. Systemic toxicity, off-target effects, delivery barriers, assay standardization, and lack of prospective OPMD validation remain major obstacles [5,195,196,197,198,204,238,239,240].

## Data Availability

No new data were created or analyzed in this study. Data sharing is not applicable to this article.

## Abbreviations

The following abbreviations are used in this manuscript:

5-ALA	5-aminolevulinic acid
8-OHdG	8-hydroxydeoxyguanosine
AKT	Protein kinase B
ATBC	Alpha-Tocopherol, Beta-Carotene Cancer Prevention Study
CAFs	Cancer-associated fibroblasts
CARET	Beta-Carotene and Retinol Efficacy Trial
CCL2	C-C motif chemokine ligand 2
circRNA	Circular RNA
COX-2	Cyclooxygenase-2
DNMT	DNA methyltransferase inhibitors
ECM	Extracellular matrix
EGFR	Epidermal growth factor receptor
EMT	Epithelial–mesenchymal transition
EPOC	Erlotinib Prevention of Oral Cancer
ER	Endoplasmic reticulum
FAK	Focal adhesion kinase histone deacetylase inhibitors
HNSCC	Head and neck squamous cell carcinoma
HPV	Human papillomavirus
hTERT	Human telomerase reverse transcriptase
IFN-γ	Interferon-gamma
IL	Interleukin
lncRNA	Long non-coding RNA
JAK	Janus kinase
LOH	Loss of heterozygosity
MAPK	Mitogen-activated protein kinase
MCP-1	Monocyte chemotactic protein-1
MDA	Malondialdehyde
MDSCs	Myeloid-derived suppressor cells
miRNA	MicroRNA
MMPs	Matrix metalloproteinases
mTOR	Mechanistic target of rapamycin
NF-κB	Nuclear factor-κB
NK	Natural killer
NSAIDs	Non-steroidal anti-inflammatory drugs
ncRNA	Non-coding RNA
OE	Oral erythroplakia
OLK	Oral leukoplakia
OLP	Oral lichen planus
OSCC	Oral squamous cell carcinoma
OSF	Oral submucous fibrosis
OPMDs	Oral potentially malignant disorders
PD-L1	Programmed death-ligand 1
PDT	Photodynamic therapy
PGE_2_	Prostaglandin E_2_
PI3K	Phosphoinositide 3-kinase
RNS	Reactive nitrogen species
ROS	Reactive oxygen species
STAT3	Signal transducer and activator of transcription 3
TGF-β	Transforming growth factor beta
Th1	T helper 1
Th17	T helper 17
Th2	T helper 2
TIMPs	Tissue inhibitors of metalloproteinases
TLR	Toll-like receptor
TNF	Tumor necrosis factor-α
Tregs	Regulatory T cells
VEGF	Vascular endothelial growth factor
YAP/TAZ	Yes-associated protein/transcriptional coactivator with PDZ-binding motif
